# Preparation, Optical, and Heat Resistance Properties of Phenyl-Modified Silicone Gel

**DOI:** 10.3390/polym17010009

**Published:** 2024-12-24

**Authors:** Xueming Chen, Xuan Wu, Chen Jin, Leiyu Hou, Shuting Zhang, Yipin Zhang, Hong Dong, Yanjiang Song, Zhirong Qu, Chuan Wu

**Affiliations:** Key Laboratory of Organosilicon Chemistry and Material Technology, College of Material, Chemistry and Chemical Engineering, Ministry of Education, Hangzhou Normal University, Hangzhou 311121, China; chenxueming@stu.hznu.edu.cn (X.C.); wuxuan187@stu.hznu.edu.cn (X.W.); jinchen@stu.hznu.edu.cn (C.J.); houleiyu@stu.hznu.edu.cn (L.H.); zhangshuting1@stu.hznu.edu.cn (S.Z.); zhangyipin@stu.hznu.edu.cn (Y.Z.); jsjj_syj@hznu.edu.cn (Y.S.); quzr@hznu.edu.cn (Z.Q.)

**Keywords:** silicone gel, phenyl-modified polysiloxane, balanced polymerization, non-equilibrium polymerization, UV radiation resistance, thermal properties

## Abstract

A series of Si-H- or Si-Vi-terminated, branched and linear oligomers containing Me_2_SiO segments were prepared by equilibrium polymerization or non-equilibrium polymerization initiated by living anions, respectively. These oligomers were used to improve the defects of concentrated crosslinking points and the high hardness of crosslinked products when using phenyltris(dimethylsiloxy)silane or 1,1,5,5-tetramethyl-3,3-diphenyl trisiloxane as crosslinking agents in the preparation of silicone gel. NMR, FT-IR, and GPC characterized the structure and molecular weight information of the prepared oligomers. The effects of equilibrium polymerization and the anionic non-equilibrium ring-opening polymerization methods on the structure of oligomers were investigated in detail, together with the structure, the molar ratio of SiH to SiVi, and the phenyl content on the thermal properties and the transmittance retention yield of the silicone gel. The introduction of phenyl groups increases the glass transition temperature of silicone gel from −121.29 °C to −117.71 °C when the phenyl content increased from 0.88 wt% to 3.17 wt%. Meanwhile, the thermal decomposition temperature of silicone gel at 10% weight loss in the N_2_ atmosphere increased from 440.5 °C to 480.0 °C. When the SiH/SiVi molar ratio is close to 1.0, the transmittance retention yield of the prepared silicone gel using Si-Vi-terminated phenyl T-shaped polysiloxane as the matrix and *α*, *ω*-dimethylsiloxyl-terminated PDMS as the crosslinking agent could reach 88.9% after 25 min of UV irradiation.

## 1. Introduction

The rapid development of network information technologies such as 5G technology, the Internet of Things, cloud computing, and big data has promoted the transition of touch screen bonding methods to full-bonding technology, which has led to the rapid development of liquid optical clear resin (OCR) or liquid optical clear adhesive (LOCA) [1]. Silicone and acrylate are the primary materials for liquid OCR/LOCA. Compared with acrylate, silicone material has more robust universality, a more substantial gap-filling ability, lower material shrinkage, better yellowing resistance, high-temperature resistance, moisture resistance, and thermal shock resistance. Liquid OCR or LOCA has been widely used in automotive display screens, medical equipment display screens, and other fields with high requirements for environmental protection testing.

Conventional silicone materials are composed of polydimethylsiloxane (PDMS). Compared with methyl, phenyl has a very high molar refractive index. After replacing the methyl group in the substituent group of PDMS with phenyl, the refractive index of polysiloxane can be increased from about 1.4000 to more than 1.5000 [2,3,4,5,6], which is a significant change for optical packaging materials. On the other hand, after the phenyl group replaces the methyl group, as the phenyl content increases, the low-temperature flexibility of PDMS will be reduced, while the high-temperature stability and the damping performance of PDMS will be significantly enhanced [7]. However, when there are a large number of Si-Ph groups in the polysiloxane molecules, as the content of Me_2_SiO segments decreases, the length of the Me_2_SiO segment also shortens accordingly. Although the mutual attraction between the two phenyl substituents in the Ph_2_SiO segment can effectively enhance intramolecular and intermolecular interactions, the introduction of phenyl groups will inevitably lead to the flexibility and mobility of the polymer chain decreases, thereby making the polymer stiffer [8].

Hydrosilylation is the primary reaction type for preparing silicone optical packaging materials because there is no low-molecular-weight material release, which will not cause pores in the packaging material itself, thus obtaining a dense and reliable packaging structure; in addition, there is no need to worry about the release of small molecules and their contamination of the device surface and interface. Hydrosilylation takes a polymer containing at least two Si-H functional groups in the molecule as the crosslinking agent and another polymer containing at least two Si-CH=CH_2_ functional groups in the molecule as the matrix. This reaction can be carried out under the catalysis of a platinum–vinyl complex (i.e., Karstedt’s catalyst) in a wide temperature ranging from room temperature to about 150 °C to obtain a three-dimensional crosslinked network. Light transmittance is an essential property for optical packaging materials composed of multi-component polymers through crosslinking reactions. When the refractive index difference between the components is controlled within 0.03, even if the amount of a component exceeds the stoichiometric ratio, the packaging material obtained by the crosslinking reaction can still have the property of optical transparency.

**Chart 1:**
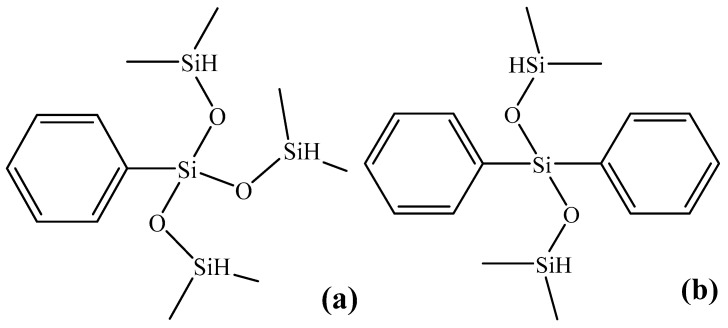
Structures of phenyltris(dimethylsiloxy)silane (**a**) and 1,1,5,5-tetramethyl-3,3-diphenyl trisiloxane (**b**).

As a typical sealant in the electronics industry, silicone materials have been successful in protecting components. In optical fiber manufacturing, a polymer matrix material and a crosslinker with a matching refractive index are essential. When phenyltris(dimethylsiloxy)silane (PTDS, Chart 1a) is used as a crosslinker for optical fibers, it can protect biased positive-intrinsic-negative (PIN’S) structures for more than 5000 h under 85/85 test conditions. In addition, due to the high boiling point of PTDS, the effect of composition changes on optical fiber performance can be minimized during the degassing process of the formulation [9]. PTDS can also be used in new olefin-based compositions to improve the high potential induced degradation (PID) risk when EVA is used in high-efficiency passivated emitters and rear-cell (PERC) bifacial module packaging materials by improving the curing response of the polymer, such as obtaining a higher MH value (the highest torque) and a lower T90 value (the time to achieve 90% torque increase) [10]. When PTDS is used as a formulation for curing rubbers such as EPDM, it can reduce VOC emissions and odor while maintaining or improving the curing characteristics of the rubber [11]. In addition, PTDS can also be used to prepare 3D printable curable silicone rubber compositions with heat humidity stabilization and self-adhesive properties [12]. PTDS can further be used in liquid silicone rubber components for the production of molded silicone rubber by injection molding, significantly reducing the cure temperature in the mold cavity to about 100 °C without reducing the cure rate and physical properties of the LSR and the molded silicone rubber material produced [13].

When a dimethylsiloxy group in the PTDS molecule is replaced with a phenyl group, 1,1,5,5-tetramethyl-3,3-diphenyl trisiloxane (TMDPTS, Chart 1b) is obtained, and it can be used as a crosslinking agent in silicone rubber systems to obtain transparent silicone rubber products with excellent thixotropic properties, heat resistance, electrical insulation properties, and weather resistance, which are widely used as protective coating agents, encapsulants or sealants for electrical/electronic equipment [14]. TMDPTS can also be used to construct high-temperature resistant thermosetting resins by hydrosilylation reaction with diethynyl aryl compounds, permitting uninterrupted function in metal-clad printed circuit board applications with comparable demanding performance or in service environments [15]. In addition, TMDPTS can be used as an adhesion promoter in the preparation of modified polyolefin resin/silicone elastomer layered articles [16], as a crosslinking agent in addition-type silicone rubber materials for LED encapsulation [17], or be used to prepare a curable oganopolysiloxane composition with excellent toughness, flexibility, and resistance to light and heat and functioned as the ideal encapsulant for a LED or a semiconductor device of superior reliability [18].

Although PTDS and TMDPTS contain phenyl groups and Si-H functional groups, they are crosslinking agents with a high refractive index and small molecular weights. When used as crosslinking agents for hydrosilylation reactions, the prepared crosslinked products have a high crosslinking density, concentrated crosslinking points, and high product hardness, which make them unable to effectively dissipate the stress impact of external forces on the packaged devices.

To overcome the defect of the high rigidity of phenyl-containing silane compounds, this study intended to introduce flexible Me_2_SiO segments into silane compounds to prepare polysiloxanes with different linear or branched structures containing active Si-H or Si-Vi bonds to improve the performance of crosslinked products and expand their application areas. In order to achieve matching refractive indices between the matrix and the crosslinking agent, this study also prepared polysiloxanes containing phenyl, vinyl, and Me_2_SiO segments as the matrix. The prepared phenyl-functionalized polymers and crosslinking agents were reacted by hydrosilylation to prepare silicone gels. The effects of different preparation methods and the phenyl contents on the thermal and optical properties of the cured silicone gel were investigated, which will guide the preparation and performance modification of phenyl optical packaging materials.

## 2. Experimental Section

### 2.1. Raw Materials

Diphenyldichlorosilane and tetrahydrofuran (THF), analytical-grade, were purchased from Sinopharm Chemical Reagent Co., Ltd. (Shanghai, China). Lithium dimethylvinylsilanol (1.0 mol/L in THF) and lithium dimethylsilanol (1.0 mol/L in THF) were kindly provided by Zhejiang Equation New Materials Co., Ltd. (Quzhou, China). Hexamethylcyclotrisiloxane (D_3_) was purchased from Shandong Dongyue Organosilicon Materials Co., Ltd. (Zibo, China). Octamethylcyclotetrasiloxane (D_4_) was purchased from Zhejiang Wynca Chemical Group Co., Ltd. (Hangzhou, China). Solid acid catalyst (HND-580) was purchased from Jiangyin Nanda Synthetic Chemical Co., Ltd. (Wuxi, China). Toluene was purchased from Hangzhou Shuanglin Chemical Co., Ltd. (Hangzhou, China). 1-Ethynyl-1-cyclohexanol (ECH) was purchased from ScienMax Inc. (Plano, TX, USA) and diluted to 0.1 wt% in toluene for easy weighing. Karstedt’s catalyst with a platinum content of 20 wt% was purchased from Heraeus Precious Metals Technology (China) Co., Ltd. (Nanjing, China) and diluted with toluene to a platinum content of 0.1 wt% for easy weighing. PTDS (UC-232), TMDPTS (UC-235), *α*, *ω*-dimethylvinylsiloxyl-terminated PDMS (UC-273-100) with a viscosity of 97.50 mPa.s at 25 °C, a *M*n of 6000 g/mol, and a vinyl content of 1.12 × 10^−4^ mol/g and *α*, *ω*-dimethylsiloxyl-terminated PDMS (UC-616-79) with a viscosity of 88.75 mPa.s at 25 °C, a *M*n of 7200 g/mol, and a hydrogen content of 2.76 × 10^−4^ mol/g, were kindly provided by Jiaxing United Chemical Co., Ltd. (Jiaxing, China). Phenyltrichlorosilane with a mass fraction of 99.5 wt%, was generously provided by Zhejiang Kaihua Synthetic Materials Co., Ltd. (Quzhou, China).

To reduce the water content in THF, an appropriate amount of sodium flakes (Na) was added to THF under a nitrogen atmosphere, and then benzophenone was added as an indicator. The mixture was heated under reflux until the solution turned bluish-purple, and then THF was collected. Except for THF, the other raw materials and reagents were used directly without further treatment.

### 2.2. Experimental Instruments

The dynamic viscosity of each liquid polysiloxane sample at 25 ± 0.1 °C and a shear rate of 30 s^−1^ was measured using a rotational viscometer (Brookfield DV2TRVTJ0, Brookfield, WI, USA). Gel permeation chromatography (GPC, PL-GPC50, Agilent, CA, USA) was used with a Styragle^®^HT2 Toluene separation column with a size of 7.8 × 300 mm. PDMS reference materials with known molecular weights (490, 6200, 15,000, 34,100, 64,800, 95,800, 219,400, 384,500, and 610,000 g/mol, American Polymer Standards Company, Mentor, OH, USA) were used as standards. Toluene was used as the mobile phase with a flow rate of 1.0 mL/min, and the column box temperature was controlled at 35 °C. The weight-average molecular weight (*M*w), number-average molecular weight (*M*n), and polydispersity index (PDI) of each polymer were tested. The ^1^H NMR spectra of the samples were measured using a nuclear magnetic resonance (NMR) instrument (AVANCE AV 400, Bruker, Karlsruhe, Germany). During the test, about 10 mg of the sample was dissolved in about 0.6 mL of deuterated chloroform (CDCl_3_) reagent without tetramethylsilane (TMS), and the relaxation time D_1_ was 5 s. The ^13^C NMR spectrum of each sample was tested using an AVANCE AV 500 nuclear magnetic resonance (NMR) instrument (Bruker, Karlsruhe, Germany). During the test, about 100 mg of the sample was dissolved in about 0.6 mL of deuterated chloroform (CDCl_3_) reagent without TMS, and the relaxation time D_1_ was 3 s. The ^29^Si NMR spectrum of each sample was tested using an AVANCE AV 500 nuclear magnetic resonance (NMR) instrument (Bruker, Karlsruhe, Germany). During the test, about 300 mg of the sample was dissolved in about 0.6 mL of deuterated chloroform (CDCl_3_) reagent without TMS, and the relaxation time D_1_ was 20 s. The products were characterized with an FT-IR spectrometer (Bruker Alpha-T, Bruker, Karlsruhe, Germany) using the KBr pellet method with a test range of 4000~400 cm^−1^ and a resolution of 4 cm^−1^. Thermal analysis was performed using a thermogravimetric (TGA) analyzer (Discovery TGA, TA Instruments, New Castle, DE, USA). The thermal decomposition properties of the samples were tested in a nitrogen atmosphere with a test temperature range of 40~800 °C, a heating rate of 20 °C/min, and a nitrogen rate of 30 mL/min. The refractive index of the samples was measured using an Abbe refractometer (WYA-2W, Shanghai INESA Physico-Optical Instrument Co., Ltd., Shanghai, China) at a test temperature of 25 ± 0.1 °C. A Netzsch DSC 214 (Netzsch, Selb, Germany) instrument was used to record the DSC curves of samples in an N_2_ atmosphere with a flow rate of 40 mL/min. To eliminate the heat history, the sample was first raised from 40 °C to 200 °C at a heating rate of 10 °C/min, then cooled from 200 °C to −150 °C at a rate of −10 °C/min, and then heated from −150 °C to 200 °C at a heating rate of 10 °C/min. The heat absorbed or released by the sample was recorded automatically to obtain the DSC curves of the cooling and heating stages.

The gelation yield (*G*_t_) of the crosslinked silicone gel was calculated from the equation *G*t = *m*_2_/*m*_1_ × 100%. The symbol *m*_1_ is the mass weight of the silicone gel sample prior to immersing it into the toluene solvent. After the sample had been immersed in the toluene for 24 h, it was taken out and dried in an oven at 80 °C for 1 h. The mass weight of the sample was then recorded as *m*_2_.

### 2.3. Preparation of Crosslinking Agents Containing Phenyl Groups and Si-H Bonds

#### 2.3.1. Si-H-Terminated T-Shaped Siloxane Oligomers (PhT-SiH)

It could be inferred from the chemical structure of PTDS shown in Chart 1a that there were three active Si-H functional groups. Therefore, this compound could be used as a trifunctional crosslinking agent for various hydrosilylation reactions [9,19,20,21,22,23,24] or could be used as a scavenger of Si free radicals initiated by active free radicals generated by dicumyl peroxide (DCP) in the preparation process of cables with insulation layers [25]. To overcome the defect of PTDS mentioned above, some flexible Me_2_SiO segments were intended to be introduced into its molecule through chemical reactions.

(1) Equilibrium polymerization

**Scheme 1:**
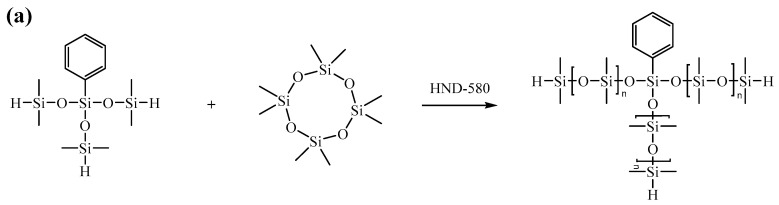

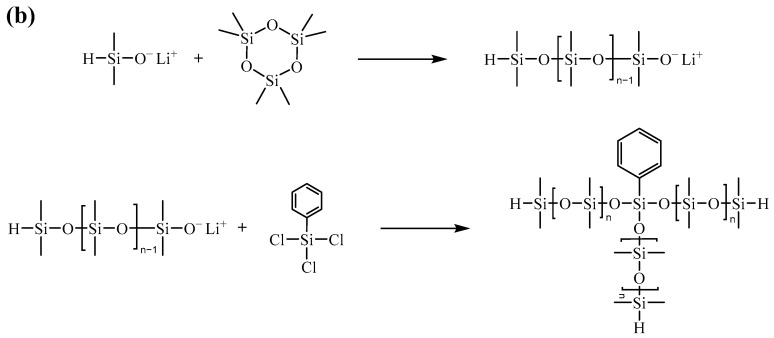
Preparation of Si-H-terminated T-shaped siloxane oligomers (PhT-SiH). (**a**) Equilibrium polymerization; (**b**) anionic non-equilibrium ring-opening polymerization.

With the use of a macroporous cation exchange resin (HND-580) as the catalyst and PTDS and D_4_ as the polymerization monomers, the Si-H-terminated T-shaped siloxane oligomer (PhT-SiH-A) was prepared using the cationic catalyzed ring-opening polymerization method, as shown in Scheme 1a. When taking Entry PhT-SiH-A6 in Table S1 of the Supporting Information as an example, the typical synthesis method of PhT-SiH-A oligomers is shown below.

In total, 133.48 g (0.45 mol) of D_4_, 33.07 g (0.1 mol) of PTDS, and 5.00 g of HND-580 (the catalyst needs to be dried at 110 °C for 3 h before use) was added to a 500 mL three-necked flask that was dry, oxygen-free, and filled with nitrogen. Then, the mixture was raised to 70 °C and reacted at this temperature for 8 h. After the reaction was completed, it was cooled down and filtered to remove the catalyst. The filtrate was subjected to reduced pressure distillation at 180 °C and −101.1 kPa for 3 h to remove low-boiling substances to collect 143.11 g of the product with a yield of 85.9%.

In changing the amount of raw materials, target polymers with different designed polymerization degrees were synthesized, and the results are shown in Table S1 of the Supporting Information.

(2) Anionic non-equilibrium ring-opening polymerization

In addition to being prepared with the equilibrium polymerization method, Si-H-terminated T-branched siloxane oligomers (PhT-SiH) were also prepared using the “living” anionic ring-opening polymerization method, as shown in Scheme 1b, with lithium dimethylsilanol as the initiator, D_3_ as the polymerization monomer, and phenyl trichlorosilane as the end-capping agent.

With the use of PhT-SiH-B6, as shown in Table S2 of the Supporting Information, as an example, the specific preparation process of PhT-SiH-B type oligomers was as follows. In total, 50.00 mL of THF was added to a 1000 mL three-necked flask that was dry, oxygen-free, and nitrogen-filled; the three-necked flask was placed in an ice bath and stirred; the temperature was controlled at about 0 °C; 270.0 mL (0.27 mol) of a 1 mol/L THF solution of lithium dimethylsilanol was added at a constant rate to the three-necked flask, which was stirred at 0 °C for 30 min after the addition; and then, the THF solution of D_3_ (by dissolving 120.0 g (0.54 mol) of D_3_ in 120.00 mL of THF solution) was added to the three-necked flask, and the temperature was raised to 25 °C for 8 h. After the reaction, 20.94 g (0.099 mol) of PhSiCl_3_ was slowly added dropwise to the three-necked flask for about 1 h. After the reaction was completed, the solution was heated under reduced pressure to remove low-boiling substances such as THF, and then LiCl was filtered out under reduced pressure. The filtrate was collected and distilled at 180 °C under a reduced pressure of −101.1 kPa for 3 h to remove low-boiling substances, and 122.22 g of the synthetic product was collected with a yield of 81.6% relative to lithium dimethylsilanol.

Changing the composition of the raw materials can synthesize different target polymers. According to Scheme 1b, a series of PhT-SiH-B oligomers with different molecular weights were prepared using the “living” anionic ring-opening polymerization method. The feeding amounts and the reaction yields are listed in Table S2 of the Supporting Information.

#### 2.3.2. Ph_2_SiO-Bridged Si-H-Terminated Linear Siloxane Oligomers (Ph_2_SiO-SiH)

There were two phenyl groups and two Si-H bonds in TMDPTS, which made TMDPTS have a refractive index of 1.5330 at 20 °C and usable as a crosslinking agent for vinyl silicone resin [26,27], a crosslinking agent for liquid-ethylene propylene diene rubber (L-EPDM) [28], or a crosslinking agent for silicone rubber [29]. The drawback of TMDPTS used in the crosslinking agent was similar to that of PTDS, and it could be overcome by introducing the flexible Me_2_SiO through chemical reactions.

(1) Equilibrium polymerization

**Scheme 2:**
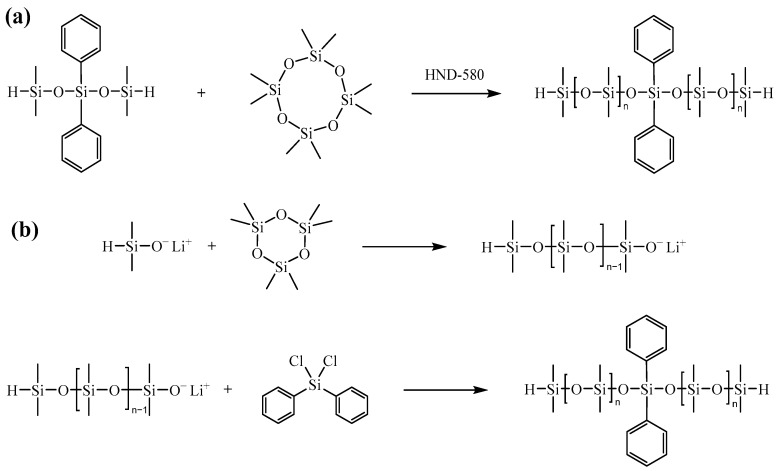
Preparation of Ph_2_SiO-bridged Si-H-terminated linear siloxane oligomers (Ph_2_SiO-SiH). (**a**) Equilibrium polymerization; (**b**) anionic non-equilibrium ring-opening polymerization.

According to Scheme 2a, HND-580 was used as a catalyst, TMDPTS and D_4_ were used as the polymerization monomers to prepare a Ph_2_SiO-bridged Si-H-terminated linear siloxane oligomer (Ph_2_SiO-SiH-A) with the cationic ring-opening polymerization method. Taking Ph_2_SiO-SiH-A6, in Table S3 of the Supporting Information, as an example, the typical synthesis route of this oligomer is shown below.

In total, 133.48 g (0.45 mol) of D_4_, 49.84 g (0.15 mol) of TMDPTS, and 5.50 g of HND-580 (dried at 110 °C for 3 h before use) was added to a 500 mL three-necked flask that was dry, oxygen-free, and filled with nitrogen. The mixture was then heated to 70 °C and reacted at this temperature for 8 h. After the reaction was completed, the temperature was cooled to room temperature to remove the catalyst by filtration. The filtrate was subjected to reduced pressure distillation at 180 °C and −101.1 kPa for 3 h to remove low-boiling substances to obtain 157.51 g of the product with a yield of 85.9%. Through changing the composition of the raw materials, Ph_2_SiO-SiH-A-type oligomers with different molecular weights were prepared using the same procedure. The results are shown in Table S3 of the Supporting Information.

(2) Anionic non-equilibrium ring-opening polymerization

Apart from the equilibrium polymerization method, polysiloxanes with a Ph_2_SiO-SiH structure were also prepared using the “living” anionic ring-opening polymerization method shown in Scheme 2b, using lithium dimethylsilanol as the initiator, D_3_ as the polymerization monomer, and diphenyldichlorosilane as the bridging agent. Taking Ph_2_SiO-SiH-B6, in Table S4 of the Supporting Information, as an example, the typical synthetic technical method of this kind of polymer is shown below.

In total, 40.00 mL of THF was added to a 500 mL dried three-necked flask that was purged with nitrogen gas. The three-necked flask was then put in an ice bath under stirring. In total, 135 mL (0.135 mol) of a 1 mol/L THF solution of lithium dimethylsilanol was then added to the three-necked flask at a constant rate. The mixture was stirred at 0 °C for 30 min after the addition. After that, the THF solution of D_3_ (dissolving 60.00 g (0.54 mol) of D_3_ in 60.00 mL of THF solution) was added to the three-necked flask for 1 h. After the addition of the THF solution of D_3_, the temperature was raised to 25 °C, and the reaction lasted at this temperature for 8 h. When the reaction was completed, 18.79 g (0.0743 mol) of Ph_2_SiCl_2_ was added slowly and reacted for about 1 h. After the condensation reaction was completed, the solution was distilled under reduced pressure to remove low-boiling substances such as THF. LiCl precipitate was removed from the reaction mixture by filtration. The collected filtrate was then subjected to reduced pressure distillation at 180 °C and −101.1 kPa for 3 h to remove low-boiling substances to obtain 69.91 g of the product with a yield of 84.8% relative to lithium dimethylsilanol. Through changing the composition of the raw materials, Ph_2_SiO-SiH-B-type oligomers with different molecular weights were prepared using the same procedure. The results are shown in Table S4 of the Supporting Information.

### 2.4. Preparation of Polymer Matrix Containing Si-Ph and Si-Vi Groups

#### 2.4.1. Si-Vi-Terminated T-Shaped Siloxane Oligomers (PhT-SiVi)

**Scheme 3:**
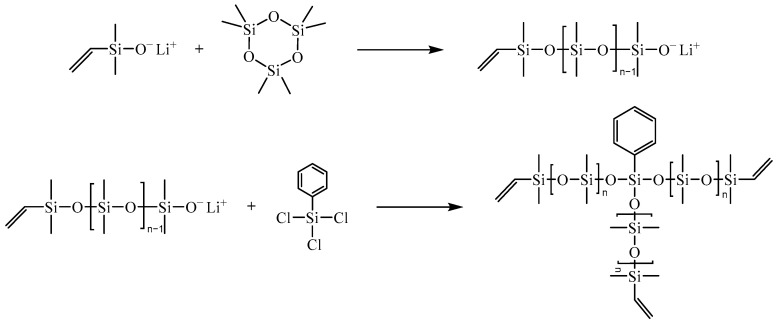
Preparation of Si-Vi-terminated T-shaped siloxane oligomers (PhT-SiVi) by “living” anionic ring-opening polymerization.

In changing the terminal group and replacing the initiator lithium dimethylsilanol with lithium dimethylvinylsilanol, the Si-Vi-terminated T-shaped siloxane oligomers (PhT-SiVi) were also prepared using the “living” anionic ring-opening polymerization method. The synthetic method is shown in Scheme 3. Taking Entry PhT-SiVi-B6 in Table S5 of the Supporting Information as an example, the typical synthetic method of this kind of oligomer is shown below.

In a dried, oxygen-free, and nitrogen-filled 1000 mL three-necked flask, 60.00 mL of THF was added, and the three-necked flask was then placed in an ice bath under stirring. After that, 270.0 mL (0.27 mol) of 1 mol/L lithium dimethylvinylsilanol in THF solution was added to the three-necked flask at a constant rate and stirred at 0 °C for 30 min after the addition. Then, the THF solution of D_3_ (by dissolving 120.00 g (0.54 mol) of D_3_ in 120.00 mL of THF solution) was added to the three-necked flask evenly. The mixture was then raised to 25 °C and reacted at this temperature for 8 h. When the reaction was completed, 20.92 g (0.099 mol) of PhSiCl_3_ was added slowly to the mixture and reacted for about 1 h. After the reaction was completed, the solution was distilled under reduced pressure to remove low-boiling substances such as THF. LiCl was precipitated and filtered out, and the filtrate was collected and distilled under reduced pressure at 180 °C and −101.1 kPa for 3 h to remove low-boiling substances to obtain 145.56 g of the product with a yield of 92.9% relative to lithium dimethylvinylsilanol. In changing the composition of the raw materials, other PhT-SiVi-type oligomers with different molecular weights were prepared using the same procedure. The results are shown in Table S5 of the Supporting Information.

#### 2.4.2. Ph_2_SiO-Bridged Si-Vi-Terminated Linear Siloxane Oligomers (Ph_2_SiO-SiVi)

**Scheme 4:**
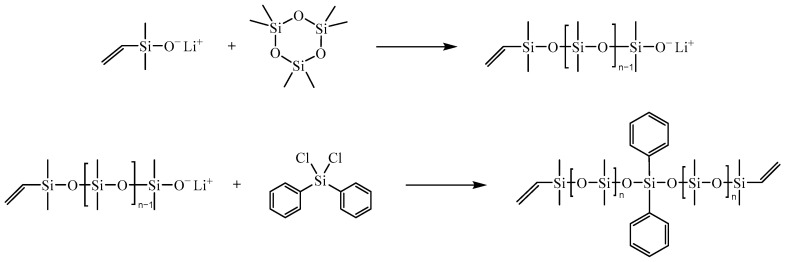
Preparation of Ph_2_SiO-bridged Si-Vi-terminated linear siloxane oligomers (Ph_2_SiO-SiVi) by “living” anionic ring-opening polymerization.

In changing the terminal groups, replacing the lithium dimethylsilanol with lithium dimethylvinylsilanol, and using Ph_2_SiCl_2_ as a bridging agent, a series of linear siloxane oligomers (Ph_2_SiO-SiVi) were prepared by anionic ring-opening polymerization. Their synthesis route is shown in Scheme 4. Taking Entry Ph_2_SiO-SiVi-B6 in Table S6 of the Supporting Information as an example, the typical preparation method of Ph_2_SiO-SiVi oligomers is shown below.

In a dried, oxygen-free, and nitrogen-filled 500 mL three-necked flask, 40.00 mL of THF was added, and the three-necked flask was then placed in an ice bath under stirring. After that, 135.0 mL (0.135 mol) of 1 mol/L lithium dimethylvinylsilanol in THF solution was added to the three-necked flask at a constant rate and stirred at 0 °C for 30 min after the addition. Then, the THF solution of D_3_ (by dissolving 60.00 g (0.27 mol) of D_3_ in 60.00 mL of THF solution) was added to the three-necked flask evenly. The mixture was then raised to 25 °C and reacted at this temperature for 8 h. When the reaction was completed, 18.74 g (0.074 mol) of Ph_2_SiCl_2_ was added slowly to the mixture and reacted for about 1 h. After the reaction was completed, the solution was distilled under reduced pressure to remove low-boiling substances such as THF. LiCl was precipitated and filtered out, and the filtrate was collected and distilled under reduced pressure at 180 °C and −101.1 kPa for 3 h to remove low-boiling substances to obtain 81.42 g of the product with a yield of 94.7% relative to lithium dimethylvinylsilanol. In changing the composition of the raw materials, other Ph_2_SiO-SiVi-type oligomers with different molecular weights were prepared using the same procedure. The results are shown in Table S6 of the Supporting Information.

### 2.5. Preparation of Phenyl Group-Functionalized Silicone Gel

#### 2.5.1. Using Dimethylvinylsiloxyl-Terminated Linear PDMS as the Matrix

In using commercially available *α*, *ω*-dimethylvinylsiloxyl-terminated PDMS (UC-273-100) or synthesized Ph_2_SiO-bridged Si-Vi group-terminated PDMS (Ph_2_SiO-SiVi) as the matrix, Si-H-terminated T-shaped siloxane oligomer (PhT-SiH) as the crosslinking agent, Pt–vinyl complex (Karstedt’s catalyst) with a platinum content of 5 × 10^−6^ (5 ppm) of the sum of the mass of the crosslinking agent and the matrix as the catalyst, and ECH with a content of 100 ppm as the inhibitor, a silicone gel sample was mixed by blending these chemicals in a mixing cup, stirring in a non-invasive material homogenizer, and removing bubbles at −99.7 kPa and 1800 rpm for 5 min. The mixed and degassed sample was then poured into a 75 mm × 10 mm × 2 mm PTFE mold and cured in an oven at 120 °C for 2 h to prepare an optically transparent silicone gel sample. Table 1 presents the structure of each oligomer used in the preparation of silicone gel, and Table 2 shows the specific dosage of each material.

#### 2.5.2. Using Si-Vi-Terminated T-Shaped Siloxane Oligomers as the Matrix

In using PhT-SiVi type siloxane oligomers as the matrix, the commercially available *α*, *ω*-bisdimethylsiloxy-terminated PDMS (UC-616-79) with a hydrogen content of 2.76 × 10^−4^ mol/g or Si-H-terminated Ph_2_SiO-bridged linear PDMS (Ph_2_SiO-SiH) as the crosslinking agent, and Karstedt’s catalyst with the same Pt content as the catalyst and ECH with the same amount as the inhibitor, optically transparent silicone gel was prepared according to the same procedure as above. The results are shown in Table 3.

## 3. Results and Discussion

### 3.1. Oligomer Structure Analysis

#### 3.1.1. PhT-SiH Oligomers

Taking Entry PhT-SiH-B6 in Table S2 of the Supporting Information as an example, the prepared Si-H-terminated T-shaped siloxane oligomer (PhT-SiH) was analyzed in the Supporting Information section by ^1^H NMR (Figure S1a), ^13^C NMR (Figure S1b), ^29^Si NMR (Figure S1c), FT-IR (Figure S1d), and GPC (Figure S1e).

As shown in Figure S1a of the Supporting Information, the signal at *δ* = 7.26 ppm was the chemical shift of the proton in C***D***Cl_3_, signals at *δ* = 0.05~0.19 ppm were the chemical shift of the protons attributed to -Si-C***H***_3_ located at side chains and terminal positions in the polymer molecule, signals at *δ* = 1.50~1.70 ppm were the chemical shift of protons in -Si-O***H*** or trace amounts of ***H***_2_O in deuterated chloroform, signals at *δ* = 4.69~4.72 ppm were the chemical shift of proton in -Si-***H***, and signals at δ = 7.41~7.73 ppm were the chemical shift of all protons on the benzene ring in -Si-Ph. According to the structural formula and ^1^H NMR spectrum of Entry PhT-SiH-B6, there was only one proton on the Si-H bond; if its integrated area was defined as 1.00, the integrated area of all protons on -Si-C***H***_3_ was 62.69. From 6n + 6 = 62.69, one can calculate that n ≈ 9, and the molecular weight of the synthesized product was determined to be *M*_NMR_ = 2413 g/mol.

From the ^13^C NMR spectrum of the sample (Figure S1b in the Supporting Information), it could be seen that *δ* = 76.71 ppm was the chemical shift of C atoms in ***C***DCl3, *δ* = 0~1.02 ppm was the chemical shift of C atoms in the terminal or sided ***C***H_3_ connected to Si-O bonds, and *δ* = 127.40~133.58 ppm was the chemical shift of C atoms in -Si-Ph. Due to the influence of the testing environment and the presence of benzene rings in the polymer, the intensity of the C atom peak became smaller, and the characteristic peaks were not pronounced, as shown in positions b and c in Figure S1b of the Supporting Information.

As shown in Figure S1c of the Supporting Information, *δ* = −6.94 ppm was the characteristic peak of Si atoms in ***Si***-H; at chemical shift *δ* = −21.96~−19.18 ppm, these signals were attributed to the characteristic peak of Si atoms in D-chain (-O-***Si***(CH_3_)_2_-O- segments), and at chemical shift *δ* = −80.82 ppm, the characteristic peak of Si atom attributed to T-segment (-O_3_***Si***-Ph) could be clearly observed.

In the FT-IR spectrum shown in Figure S1d of the Supporting Information, the wave number at 2962 cm^−1^ was the C-H stretching vibration peak in -Si-CH_3_; the wave number at 2127 cm^−1^ was the stretching vibration peak of -Si-H; the wave numbers at 1258 cm^−1^ and 787 cm^−1^ were the characteristic peaks of -Si-CH_3_; the wave number at 1015 cm^−1^ was the characteristic peak of Si-O-Si; and the wave number at 666 cm^−1^ was the characteristic peak of -Si-Ph.

In the GPC curve of the polymer sample (Figure S1e of the Supporting Information), only one sample peak was observed, which clearly confirms the characteristics of high-molecular-weight polymers and shows a typical distribution characteristic. The weight-average molecular weight (*M*w) of the measured sample was 2100 g/mol, the number-average molecular weight (*M*n) was 1100 g/mol, and the polydispersity index (PDI) was about 1.91. Since the synthesized target product contains phenyl and has a T-shaped structure, its molecular weight distribution is broad with a large PDI value.

#### 3.1.2. Ph_2_SiO-SiH Oligomers

Taking Entry Ph_2_SiO-SiH-B18 in Table S4 of the Supporting Information as an example, the prepared Ph_2_SiO-bridged Si-H-terminated linear siloxane oligomer (Ph_2_SiO-SiH) was analyzed in detail in the Supporting Information section by ^1^H NMR (Figure S2a), ^13^C NMR (Figure S2b), ^29^Si NMR (Figure S2c), FT-IR (Figure S2d), and GPC (Figure S2e). ^1^H NMR determined the degree of polymerization of this polymer to be *n* = 18, and the molecular weight calculated by ^1^H NMR was *M*_NMR_ = 3049 g/mol. The *M*w of the polymer was determined to be 4800 g/mol, *M*n was 2500 g/mol, and PDI was about 1.92 by GPC.

#### 3.1.3. Si-Vi-Terminated T-Shaped Siloxane Oligomers (PhT-SiVi)

Taking Entry PhT-SiVi-B15 in Table S5 of the Supporting Information as an example, the prepared Si-Vi terminated T-shaped siloxane oligomer (PhT-SiVi) was analyzed in detail in the Supporting Information section by ^1^H NMR (Figure S3a), ^13^C NMR (Figure S3b), ^29^Si NMR (Figure S3c), FT-IR (Figure S3d), and GPC (Figure S3e). ^1^H NMR determined the degree of polymerization of this polymer to be *n* = 19, and the molecular weight calculated by ^1^H NMR was *M*_NMR_ = 4515 g/mol. The *M*w of the polymer was determined to be 5500 g/mol, *M*n was 3300 g/mol, and PDI was about 1.67 by GPC.

#### 3.1.4. Ph_2_SiO-SiVi Oligomers

Taking Entry Ph_2_SiO-SiVi-B18 in Table S6 of the Supporting Information as an example, the prepared Ph_2_SiO-bridged Si-Vi-terminated linear siloxane oligomer (Ph_2_SiO-SiVi) was analyzed in detail in the Supporting Information section by ^1^H NMR (Figure S4a), ^13^C NMR (Figure S4b), FT-IR (Figure S4c), and GPC (Figure S4d). ^1^H NMR determined the degree of polymerization of this polymer to be *n* ≈ 23, and the molecular weight calculated by ^1^H NMR was *M*_NMR_ = 3777 g/mol. The *M*w of the polymer was determined to be 5700 g/mol, *M*n was 3600 g/mol, and PDI was about 1.58 by GPC.

### 3.2. Effect of Preparation Method on Oligomer Structure

#### 3.2.1. Polymers Containing Si-H and Si-Ph Groups

The analytical results of the Si-H terminated T-shaped siloxane crosslinking agent (PhT-SiH) prepared either with the cation-catalyzed equilibrium polymerization method or the “active” anion-catalyzed D_3_ non-equilibrium polymerization method are shown in Tables S1 and S2 of the Supporting Information. The analytical results of the Ph_2_SiO-SiH structure crosslinking agents prepared with either the cation-catalyzed equilibrium polymerization method or the “active” anion-catalyzed D_3_ non-equilibrium polymerization method are shown in Tables S3 and S4 of the Supporting Information.

In comparing results presented in Tables S1 with S2 and Tables S3 with S4 of the Supporting Information, it can be seen that compared with cationic ring-opening polymerization, the crosslinking agents containing Si-H and Si-Ph groups synthesized by anionic polymerization have the characteristics of a more narrow molecular weight distribution, lower viscosity, and higher yield. With the increase in molecular weight, the PDI value and viscosity of polysiloxanes tend to increase while the refractive index gradually decreases. Cationic ring-opening polymerization formed a large number of cyclic oligomers during the polymerization process, the molecular weight distribution of the product became more expansive, the molecular weight was difficult to control accurately, and the polymer chain segment distribution was uneven. The “living” anionic ring-opening polymerization method could avoid the formation of a large number of cyclic oligomers and could achieve controllable synthesis of the target polymer. It was suitable for the ring-opening polymerization of cyclosiloxanes with significant ring tension. The obtained product had a narrow molecular weight distribution, controllable reaction kinetics, and a single-product component.

#### 3.2.2. Polymers Containing Si-CH=CH_2_ and Si-Ph Groups

As shown in Table S5 of the Supporting Information, the degree of polymerization (*n*_exptl_) of the Si-Vi-terminated T-shaped siloxane oligomers prepared by “living” anionic ring-opening polymerization using the method shown in Scheme 3 deviates slightly from the designed value (*n*_desn_). The PDI values of the prepared polymers do not exceed 1.70, showing a narrow distribution characteristic. As the molecular weight or degree of polymerization of the polymer increases, the viscosity of the polymer gradually increases. Since the polymer molecule contains only one phenyl group, as the number of repeated Me_2_SiO segments introduced on each branch of the polymer molecule increases from 7 to 28, the refractive index of the polymer decreases from 1.4200 to 1.4091. As can also be seen from Table S5, the yield of the Si-Vi-terminated T-shaped siloxane oligomers prepared by “living” anionic ring-opening polymerization was higher than 90%.

The experimental results of the preparation of linear siloxane oligomers bridged by a Ph_2_SiO unit and terminated by Si-Vi groups with the “living” anionic ring-opening polymerization method (Table S6 of the Supporting Information) show that the PDI values of these polymers were all less than 1.60. Since each polymer molecule contains two phenyl functional groups with a high molar refractive index, the Ph_2_SiO-SiVi polymers with a similar number-average molecular weight have a higher refractive index than the PhT-SiVi polymers. In addition, it can be found from Table S6 that since the Ph_2_SiO-SiVi polymer uses linear Ph_2_SiCl_2_ as a bridging agent, the intermediate Me_2_ViSiO-(Me_2_SiO)_n_-Me_2_SiOLi terminated by the sizeable steric hindrance Me_2_ViSiO_1/2_- group was more likely to undergo condensation reaction with Si-Cl in the Ph_2_SiCl_2_ molecule. Hence, the yield of the Ph_2_SiO-SiVi polymer was somewhat higher (all higher than 93%) than that of the T-shaped siloxane oligomer (PhT-SiVi) prepared using PhSiCl_3_ as a bridging agent with a yield ranging from 91.4% to 92.9%.

### 3.3. Effect of Oligomer Structure on the Properties of Silicone Gel

#### 3.3.1. With Si-Vi-Terminated Linear PDMS as the Matrix

##### With Me_2_ViSiO_1/2_-Terminated PDMS (UC-273-100) as the Matrix

**Figure 1:**
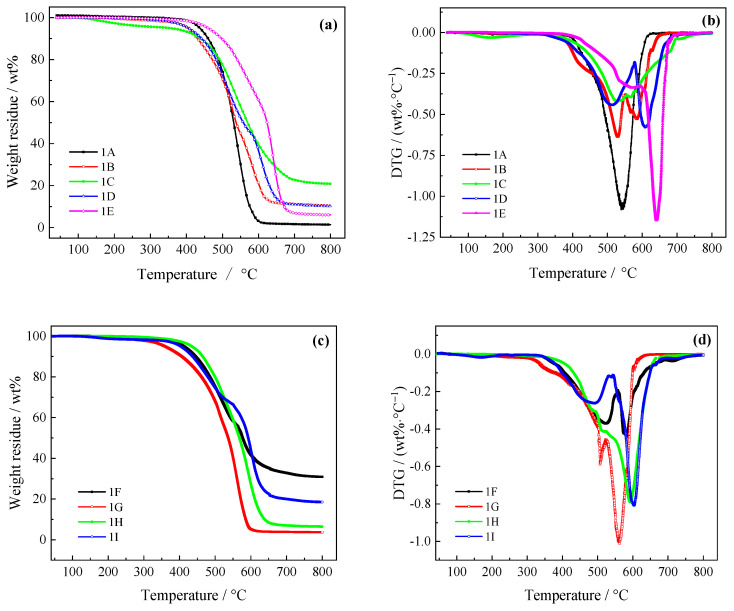
TGA curves (**a**,**c**) and DTG curves (**b**,**d**) of silicone gels prepared using Me_2_ViSiO_1/2_-terminated PDMS (UC-273-100) or Ph_2_SiO-SiVi as the matrix in N_2_ atmosphere.

In using phenyl-free *α*, *ω*-dimethylvinylsiloxy-terminated PDMS (UC-273-100) as the matrix and siloxane oligomers of different molecular weights with a PhT-SiH structure prepared by cationic ring-opening polymerization or anionic polymerization as the crosslinking agent, the two components were cured by hydrosilylation reaction, and the cured silicone gel was subjected to a gelation yield test and thermal decomposition performance test. The results are collected in Table 2 and Table 4, as well as in Figure 1a,b.

As can be seen from Table 2, the gelation yield of the silicone gel prepared using the Si-H-terminated T-shaped PDMS with the smallest molecular weight as the crosslinking agent was the highest, reaching 99.3% (Entry 1C), indicating that the crosslinking agent had high reactivity and formed an effective crosslinked network with the linear dimethylvinylsiloxyl-terminated PDMS under the action of platinum catalyst. It could also be seen from Table 2 that when the molecular weight of the crosslinking agent was similar, the gelation yield of the silicone gel (Entry 1D) cured using the PhT-SiH-structured siloxane oligomer (PhT-SiH-B15) obtained by anionic polymerization as the crosslinking agent was lower than the gelation yield of the silicone gel (Entry 1B) cured using the product of cationic ring-opening polymerization (PhT-SiH-A10) as the crosslinking agent. For the PhT-SiH-structured siloxane oligomers obtained by anionic polymerization, when they were used as the crosslinking agent, as the degree of polymerization of the Me_2_SiO segments in the polymer molecule increased, the polymer molecular weight gradually increased, and the phenyl content gradually decreased accordingly; as the molecular weight of the branched structure crosslinking agent (PhT-SiH) increased, the content of Si-H groups contained in the crosslinking agent decreased, and the degree of reaction between the branched structure crosslinking agent (PhT-SiH) and the linear *α*, *ω*-dimethylvinylsiloxyl-terminated PDMS decreased, resulting in a significant decrease in the gelation yield of the silicone gel. As the molecular weight of the branched crosslinking agent (PhT-SiH) increased, the molecular weight of the silicone gel also increased accordingly. Therefore, from Entry 1C to Entry 1E, the thermal properties of each silicone gel sample in nitrogen were significantly improved as the molecular weight of the crosslinker increased.

The gelation yield of the silicone gel obtained by the hydrosilylation of the crosslinking agent prepared by cationic catalytic equilibrium polymerization and linear *α*, *ω*-dimethylvinylsiloxyl-terminated PDMS (UC-273-100) increased slightly with the decrease in phenyl content or the increase in crosslinking molecular weight. However, the thermal properties decreased with the decrease in phenyl content. As can be seen from Figure 1a,b, whether the crosslinking agent was prepared through an anionic polymerization method or an equilibrium polymerization method, the temperature at 5% weight loss (T_5%_) of the silicone gel prepared with the PhT-SiH structure siloxane oligomer as a crosslinking agent in N_2_ atmosphere was more significant than that at 350 °C. When PhT-SiH-structured oligomers with an average molecular weight (*M*n) of 1100 g/mol prepared by anionic polymerization were used as the crosslinking agent, the prepared silicone gel (Entry 1C) had the highest residual carbon yield at 800 °C, reaching about 20.76 wt%. At the same time, the DTG curve of the silicone gel (Figure 1b and Table 4) shows that it had a maximum thermal degradation rate of about −0.40 wt%/°C in the temperature range of 534.3~566.5 °C, which was significantly lower than that of other samples. This behavior may be because the sample was prepared using PhT-SiH-B6 as the crosslinking agent, which has a regular structure, a small molecular weight, and a high hydrogen content and was prepared by non-equilibrium polymerization. The crosslinked product obtained with this crosslinking agent and the linear polymer UC-273-100 might have a denser structure and the highest gelation yield, and therefore had the best thermal properties.

##### With Ph_2_SiO-SiVi Oligomers as the Matrix

The experimental data of silicone gel prepared using *α*, *ω*-bisdimethylvinylsiloxyl-terminated PDMS containing Ph_2_SiO unit as the matrix and Si-H terminated T-shaped PDMS prepared either by equilibrium polymerization or anionic “living” non-equilibrium polymerization as the crosslinking agent are shown in Table 2 and Table 4 and Figure 1c,d.

It can be seen from Table 2 that the Si-H-terminated T-shaped PDMSs with *M*n of 1100 and 3000 g/mol prepared with the anionic living polymerization method could function as the crosslinking agents for silicone gels with higher gelation yield (98.5% for Entry 1H and 98.8% for Entry 1G) by reacting with a Ph_2_SiO-SiVi oligomer with an *M*n of 1500 g/mol or 3300 g/mol, respectively, indicating that these polymers prepared using “living” anionic non-equilibrium polymerization technology have high reactivity. When the molecular weight of the crosslinking agent and the matrix further increased, the gelation yield of the prepared silicone gel dropped significantly to only 77.8% (Entry 1I in Table 2). This result might be due to the hydrosilylation reaction of these two polymers, which was significantly affected by the steric hindrance of the high-molecular-weight polymer. Under such circumstances, the degree of hydrosilylation reaction was significantly reduced, resulting in a relatively relaxed polymer network structure. Although the molecular weight of the PhT-SiH oligomer prepared with the equilibrium polymerization process was not too much, when it was used as the crosslinking agent for the Ph_2_SiO-SiVi oligomer with a smaller molecular weight, the gelation yield of the silicone gel prepared was not high, only 85.7% (Entry 1F). This result might be attributed to the disorganized structure of the branched polymer prepared by equilibrium polymerization, which resulted in poor hydrosilylation reaction activity.

When comparing Entry 1F and Entry 1G, it could be found that although each of these two samples used Ph_2_SiO-SiVi as the matrix, Entry 1G used Si-H-terminated T-shaped PDMS with a smaller molecular weight prepared by “living” anionic polymerization as the crosslinking agent. In contrast, Entry 1F used Si-H-terminated T-shaped PDMS with a larger molecular weight prepared by equilibrium polymerization as the crosslinking agent. The gelation yield of sample Entry 1F was significantly lower than that of Entry 1G, indicating that the polymer network structure formed in Entry 1F was more relaxed. This result might be because the number of repeated Me_2_SiO segments contained in each arm of the PhT-SiH crosslinking agents prepared by equilibrium polymerization is not the same, resulting in an irregular molecular structure. On the other hand, the molecular weight of the crosslinking agent in Entry 1F was also more significant than that of the Si-H-terminated T-shaped PDMS prepared using the non-equilibrium polymerization process used in Entry 1G, resulting in a more considerable steric hindrance to overcome when sampling Entry 1F forms a crosslinked network. Hence, the gelation yield of this sample was lower.

The thermal properties of these silicone gels in N_2_ are shown in Table 4 and Figure 1c,d. Since these silicone gels used Ph_2_SiO-SiVi oligomers as the matrix, the thermal properties of these silicone gels in the N_2_ atmosphere were generally better than those prepared with vinyl-terminated linear polymers without the Ph_2_SiO unit. The gelation yield of the silicone gel prepared using PhT-SiH oligomers prepared by equilibrium polymerization as a crosslinking agent and that using Ph_2_SiO-SiVi oligomer with the smallest molecular weight as a matrix was relatively small. However, due to the higher molecular weight of the crosslinking agent, the crosslinking agent itself has good heat resistance. Therefore, the 10% weight loss temperature of the Entry 1F sample in the N_2_ atmosphere was higher than that of Entry 1G, even though the gelation yield of Entry 1G reached 98.8%. The data in Table 2 and Table 4 show that the factors affecting the thermal properties of silicone gels were very complicated and were related to both the density of the silicone gel network and the molecular weight of the crosslinking agent and the matrix.

It could also be seen from Table 4 that when the PhT-SiH-B-type oligomer prepared by “living” anionic polymerization was used as the crosslinking agent, and the Ph_2_SiO-SiVi oligomer was used as the matrix, the gelation yield of the prepared silicone gel decreased as the molecular weight of the crosslinking agent and the matrix increased. However, the thermal properties of the cured silicone gel decreased with the increase in the molecular weight of the crosslinking agent and the matrix. Although silicone gels with lower molecular weights contained higher amounts of phenyl groups, their thermal properties were not as good as those of silicone gels with lower phenyl content but larger molecular weights. These results show that although both phenyl content and molecular weight influenced the thermal properties of polymers, for high-molecular-weight polymers, the impact of molecular weight on the thermal properties of polymers was more significant than that of phenyl content.

#### 3.3.2. With Si-Vi-Terminated Branched Polymer as the Matrix

##### Using *α*, *ω*-Me_2_HSiO_1/2_-Terminated Linear PDMS (UC-616-79) as the Crosslinking Agent

**Figure 2:**
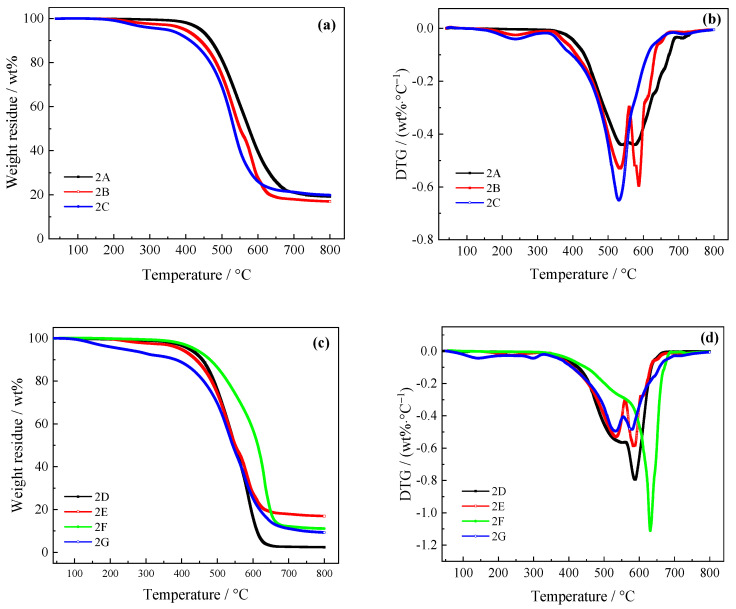
TGA curves (**a**,**c**) and DTG curves (**b**,**d**) of silicone gels prepared using PhT-SiVi as the matrix in N_2_ atmosphere.

The gelation yields and thermal properties of the silicone gel prepared using the PhT-SiVi oligomer prepared by anionic non-equilibrium polymerization as the matrix and commercially available *α*, *ω*-dimethylsiloxy-terminated PDMS (UC-616-79) as the crosslinking agent are shown in Table 3 and Figure 2a,b.

It can be seen from Table 3 that when the molecular weight of the PhT-SiVi oligomer increased, the phenyl content in the silicone gel gradually decreased. At the same time, the molar ratio of Si-H/Si-Vi in the silicone gel gradually increased, and the gelation yields of the cured silicone gel were between 82.1% and 87.9%. When comparing the data in Table 4 and Figure 2a,b, it could be found that the thermal properties of the silicone gel gradually decreased as the phenyl content decreased. For example, the *T*_10%_ value of sample Entry 2A in N_2_ was 471.8 °C. When the molecular weight of the PhT-SiVi oligomer reached 5400 g/mol (Entry 2C), the *T*_10%_ value of this kind of silicone gel in N_2_ decreased to 411.4 °C, its MRDT value was 531.1 °C, and its TDR value was −0.65 wt%/°C.

##### Using Ph_2_SiO-SiH Oligomers as the Crosslinking Agent

The gelation yields of the silicone gels prepared by hydrosilylation reaction using Ph_2_SiO-SiH oligomers prepared by either equilibrium polymerization (Entries 2D and 2E) or anion-catalyzed non-equilibrium polymerization (Entries 2F and 2G) as a crosslinking agent and PhT-SiVi oligomers prepared by anion-catalyzed non-equilibrium polymerization as the matrix are presented in Table 3. The thermal properties of these silicone gels in N_2_ are shown in Table 5 and Figure 2c,d.

It can be found from Table 3 that when the Ph_2_SiO-SiH oligomers prepared with the equilibrium polymerization method were used as the crosslinking agent, as the molecular weight of PhT-SiVi oligomers and that of the crosslinking agents increased, the gelation yields of the silicone gels gradually decreased from 94.1% (Entry 2D) to 91.5% (Entry 2E). This result occurred because as the molecular weight of the polymer participating in the reaction increased, the entanglement of the polymer segments intensified, which resulted in an increase in the steric hindrance and the inability of the Si-H bond and the Si-Vi bond to react to form a highly crosslinked polymer network structure. When comparing Entry 2D and Entry 2F, it could be seen that although the molecular weight of PhT-SiVi oligomer increased, the gelation yields of the silicone gels formed by the crosslinking agent prepared with the “living” anion polymerization process participating in the curing reaction reached 99.6%, which indicates that the Ph_2_SiO-SiH oligomers prepared with the “living” anionic polymerization process had higher reactivity than the Si-H oligomers of similar structure and molecular weight prepared with the equilibrium polymerization method. However, when the molecular weight of the Si-H oligomer increased, the gelation yields of the silicone gel containing a small-molecular-weight PhT-SiVi oligomer would decrease (Entry 2G).

As shown in Table 5 and Figure 2c,d, the silicone gel (Entry 2F) prepared with the PhT-SiVi oligomer with the most significant molecular weight as the matrix and Ph_2_SiO-SiH oligomer prepared by “living” anionic polymerization as the crosslinking agent exhibited the best heat resistance. Its *T*_10%_ value in N_2_ reached 480 °C, which was also the highest among the silicone gel samples investigated in Table 5. It could also be seen from Table 5 and Figure 2c,d that the thermal properties of these silicone gel samples were closely related to the gelation yield of silicone gel. As the gelation yield increased, the thermal properties of silicone gel improved.

Compared with the silicone optical adhesive sample reported in the literature [30] with a maximum *T*_5%_ value of 362 °C, most of the silicone gel samples prepared in this manuscript exhibited better thermal stability, as shown in Table 4 and Table 5. The superior thermal stability of these silicone gel samples might be related to the T-shaped structure contained in them, whether these T-shaped structures resulted from the matrix or the crosslinking agent significantly increased the crosslinking density of silicone gels and their thermal stability.

#### 3.3.3. Structure Analysis of Silicone Gels

**Figure 3:**
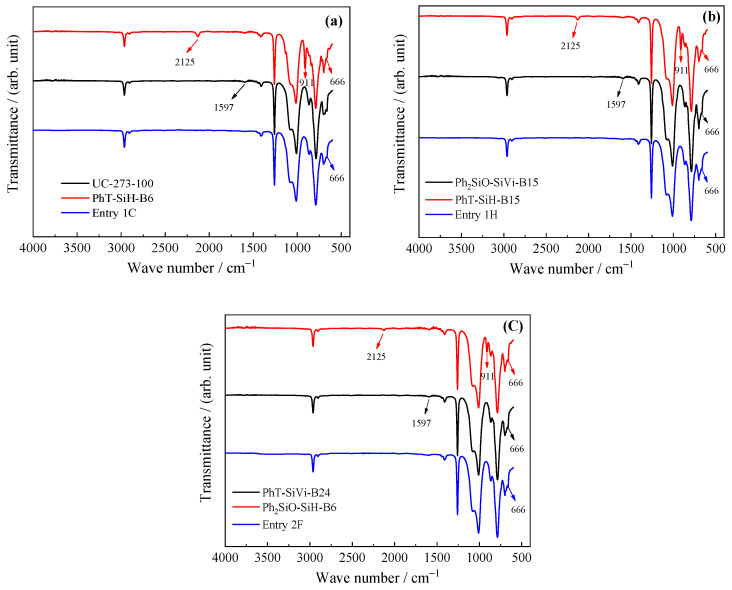
FT-IR spectra of the silicone gels, the matrix, and its crosslinking agents. (**a**) Entry 1C; (**b**) Entry 1H; (**c**) Entry 2F.

The FT-IR spectra of the silicone gel samples of Entry 1C and Entry 1H in Table 2 and Entry 2F in Table 3 as well as the matrix and crosslinking agent constituting the silicone gel are shown in Figure 3. The wave numbers at 2125 cm^−1^ and 911 cm^−1^ were the stretching vibration absorption peaks of Si-H in the crosslinking agent, 1597 cm^−1^ was the stretching vibration absorption peak of Si-CH=CH_2_ in the matrix, and 666 cm^−1^ was the characteristic peak of Si-Ph in the crosslinking agent and silicone gel samples. As shown in Figure 3a, after the silicone gel (Entry 1C) formed through the hydrosilylation reaction, the Si-H absorption peak at 2125 cm^−1^ from the crosslinking agent (PhT-SiH-B6) and the Si-CH=CH_2_ absorption peak at 1597 cm^−1^ from the matrix (UC-273-100) disappeared, while the absorption peak of Si-CH_2_-CH_2_-Si was not observed in the FT-IR spectrum of Entry 1C, which might be due to the low content of this functional group. The FT-IR spectra of the Entry 1H and Entry 2F samples and their corresponding matrix and crosslinking agent components in Figure 3b,c show similar change trends.

#### 3.3.4. DSC Analysis

**Figure 4:**
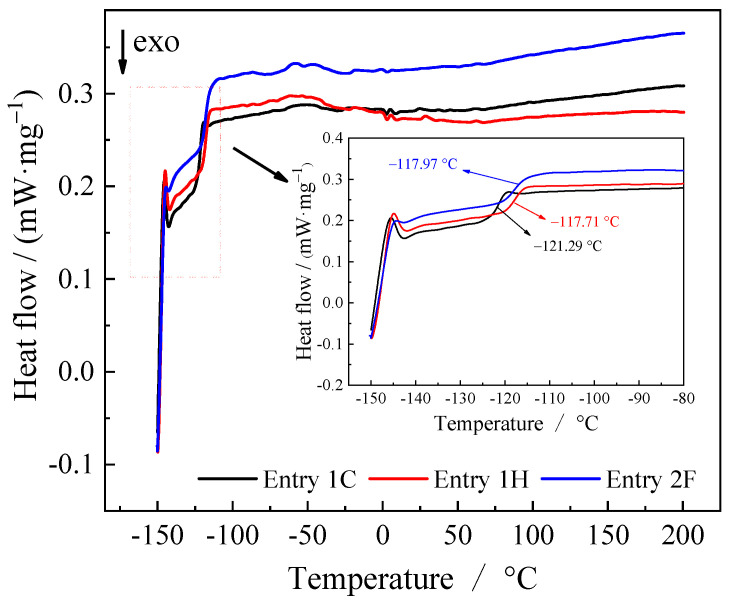
DSC curves of silicone gel samples Entry 1C, Entry 1H, and Entry 2F in N_2_ atmosphere.

The DSC curves of silicone gel samples Entry 1C, Entry 1H, and Entry 2F are shown in Figure 4. In the DSC heating curve shown in Figure 4, no obvious melting process is observed for these three samples. From the local magnified curve, it can be clearly observed that each sample had a noticeable glass transition temperature (*T*g). The glass transition temperature of Entry 1C is −121.29 °C, the glass transition temperature of Entry 1H is −117.71 °C, and the glass transition temperature of Entry 2F is −117.97 °C. As shown in Table 2 and Table 3, the phenyl contents in Entry 1C, Entry 1H, and Entry 2F were 0.88 wt%, 3.17 wt%, and 2.85 wt%, respectively. The glass transition temperature of PDMS without phenyl was close to −124 °C [31]. It could be found that with the increase in the phenyl mass fraction in silicone gel samples, the glass transition temperature of the silicone gel increased with the increase in phenyl content. This shows that the higher the content of rigid phenyl groups introduced into the silicone gel, the more severely the movement and flexibility of the molecular chain segments of silicone gel were constrained.

### 3.4. Effects of the Structures of the Matrix and Crosslinking Agents and the Phenyl Content on the UV Resistance of Silicone Gels

#### 3.4.1. Silicone Gels Without Ph_2_SiO Unit

**Figure 5:**
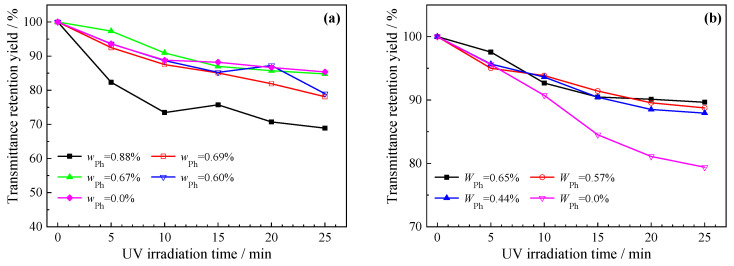
Effect of phenyl content on the radiation resistance of silicone gel. (**a**) The hydrosilylation product of a Si-H-terminated T-shaped crosslinking agent and *α*, *ω*-bisdimethylvinylsiloxyl-terminated PDMS (UC-273-100); (**b**) the hydrosilylation product of *α*, *ω*-dimethylsiloxyl-terminated PDMS (UC-616-79) and a Si-Vi-terminated T-shaped polymer.

Although introducing phenyl groups into PDMS can increase the refractive index of the material and the phenyl group can absorb ultraviolet rays and has a specific radiation resistance, the material may still turn yellow as the phenyl content increases and the irradiation time increases [32], resulting in a decrease in the optical properties. In order to investigate the effect of phenyl content on the radiation resistance of various silicone gels, PhT-SiH with different phenyl contents prepared with the equilibrium polymerization process was first used as a crosslinking agent, and *α*, *ω*-dimethylvinylsiloxyl-terminated PDMS (UC-273-100) was used as the matrix to prepare silicone gels with different phenyl contents according to the dosage of each component in Entries 3A~3E in Table 6. In Entry 3E, when MeSiCl_3_ was used instead of PhSiCl_3_ as the bridging agent to prepare the Si-H-terminated polymer, the phenyl content in the prepared crosslinking agent (MeT-SiH-A33) was zero. At the same time, commercially available *α*, *ω*-dimethylsiloxyl-terminated PDMS (UC-616-79) was also used as a crosslinking agent. PhT-SiVi oligomers with different phenyl contents prepared by “living” anionic polymerization were used as the matrix. Silicone gels with different phenyl contents were prepared according to the amounts of each component in Entries 3F~3I in Table 6. In Entry 3I, when MeSiCl_3_ was used instead of PhSiCl_3_ as the bridging agent to prepare the Si-Vi-terminated matrix, the phenyl content in the prepared matrix (MeT-SiVi-B26) was zero, and the phenyl content in the silicone gel prepared by hydrosilylation was also zero. Each silicone gel sample obtained by hydrosilylation reaction was placed in a UV curing box with a power supply of 2.2 kW and a UV lamp power of 120 mW, and the distance between the sample and the UV lamp was about 10 cm. Each silicone gel sample was irradiated in the UV box for a certain period (from 5 min to 25 min) and then taken out. The transmittance of each UV irradiated sample was tested, and the transmittance retention yield of each silicone gel sample was calculated, that is, the ratio of the transmittance of the UV irradiated sample to the transmittance of the initial sample without UV irradiation. The results are shown in Figure 5a,b.

As shown in Figure 5a, when linear *α*, *ω*-dimethylvinylsiloxyl-terminated PDMS (UC-273-100) was used as the matrix and T-shaped Si-H-terminated PDMS prepared by equilibrium polymerization was used as the crosslinking agent, as the molecular weight of the T-shaped Si-H-terminated PDMS increased, the phenyl content gradually decreased, and the transmittance retention yield of the crosslinked silicone gel sample showed a downward trend with the increase in phenyl content. However, in Figure 5b, when linear *α*, *ω*-dimethylsiloxyl-terminated PDMS (UC-616-79) was used as the crosslinking agent, and the SiVi-terminated T-shaped polymer prepared with the “living” anion polymerization process was used as the matrix, the transmittance retention yield of the cured silicone gel showed a slow downward trend with the increase in phenyl content, indicating that in this case, increasing the phenyl content helped to improve the UV irradiation resistance of the silicone gel.

As can be seen from Table 6, in Entries 3A~3E, the molar ratio of Si-H to Si-Vi was much higher than 1, while in Entries 3F~3I, the molar ratio of SiH to SiVi was close to 1, so each sample in Figure 5a contained more residual Si-H bonds. Si-H bonds were active functional groups. Under the action of high-energy rays, Si-H groups were prone to the homolytic cleavage and formation of free radicals. These highly active free radicals might undergo various reactions such as recombination, disproportionation, and hydrogen extraction [33]. The absorption of photons by the remaining unsaturated bonds in the silicone gel would lead to further photochemical reactions and ultimately cause the degradation of the crosslinked PDMS by causing intermolecular and intramolecular rearrangements [33], resulting in a decrease in transmittance.

When comparing Entry 3E in Figure 5a with Entry 3I in Figure 5b, it could be found that neither of these two samples contains phenyl groups; that is, the transmittance of the silicone gel formed by the hydrosilylation reaction had nothing to do with the phenyl groups. However, since the excess Si-H content in Entry 3E was higher than that in Entry 3I, the decrease in transmittance of sample Entry 3E was about 10% greater than that of sample Entry 3I.

#### 3.4.2. Silicone Gels Containing Ph_2_SiO Unit

**Figure 6:**
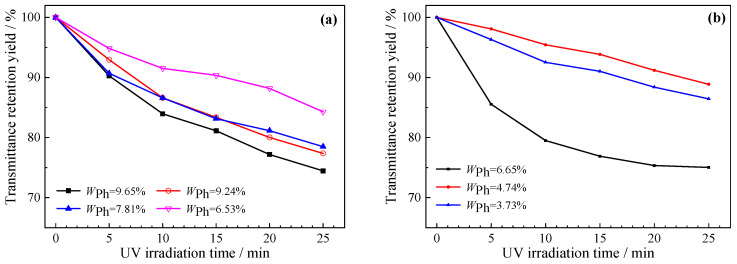
Effects of SiH/SiVi ratio and phenyl content on transmittance of Ph_2_SiO unit-bridged silicone gel. (**a**) Ph_2_SiO-SiVi-B6 as the matrix, PhT-SiH with different molecular weights prepared by equilibrium polymerization as the crosslinking agent; (**b**) PhT-SiVi-B6 as the matrix, Ph_2_SiO-SiH with different molecular weights prepared by “living” anionic polymerization as the crosslinking agent.

The change in the light transmittance retention yield of silicone gel prepared with *α*, *ω*-dimethylvinylsiloxyl-terminated PDMS (Ph_2_SiO-SiVi-B6) bridged with a Ph_2_SiO unit as the matrix and Si-H-terminated phenyl T-shaped PDMS (PhT-SiH) with different polymerization degrees as the crosslinking agent with the UV irradiation time is shown in Figure 6a. The change in the light transmittance retention yield of silicone gel prepared with *α*, *ω*-dimethylvinylsiloxyl-terminated phenyl T-shaped PDMS (PhT-SiVi-B6) as the matrix and *α*, *ω*-dimethylsiloxyl-terminated PDMS bridged with an Ph_2_SiO unit with different polymerization degrees (Ph_2_SiO-SiH) prepared by “living” anionic polymerization as the crosslinking agent with UV irradiation time is shown in Figure 6b.

As shown in Figure 6a, as the UV irradiation time increases, the transmittance retention yield of each silicone gel sample gradually decreases. As the phenyl content in the cured product increased, the transmittance retention yield of the cured product also decreased synchronously. After 25 min of UV irradiation, the transmittance retention yield of the sample with a phenyl content of 6.53 wt% reached more than 85%. In the four silicone gel samples examined in Figure 6a, the molar ratio of SiH/SiVi was between 0.24 and 0.35, which indicates that the crosslinked silicone gel samples still contained residual Si-CH=CH_2_ groups. Due to the high ultraviolet energy generated by UV light, after long-term irradiation, the residual Si-CH=CH_2_ in these silicone gel samples could also undergo photochemical reactions, causing the silicone gel to age and discolor, resulting in increased absorption. For example, even though sample Entry 4A contained 9.65 wt% of phenyl functional groups, after 25 min of UV irradiation, the transmittance retention yield of the silicone gel was only about 75%.

In each sample shown in Figure 6b, the SiH/SiVi molar ratio was close to 1. In the silicone gel generated by the hydrosilylation reaction, the remaining Si-H or Si-Vi was relatively small, which means that the chemical structure of the silicone gel was relatively stable, so the adverse effect of the residual Si-H or Si-CH=CH_2_ functional groups on the transmittance of these silicone gel samples could be ignored. As shown in Figure 6b, when the phenyl content was lower than 4.80 wt%, after 25 min of UV irradiation, the transmittance retention yields of samples Entry 4F and Entry 4G reached 88.9% and 86.4%, respectively, while the transmittance retention yield of the sample with a phenyl mass fraction of 6.65 wt% (Entry 4E) was only 75.0%, indicating that increasing the phenyl content in the silicone gel sample could not improve the UV radiation resistance of the silicone gel although the phenyl functional group can absorb the energy of UV rays.

When comparing Table 6 and Table 7, as well as Figure 5b and Figure 6b, it can be seen that when the SiH/SiVi molar ratio in the silicone gel was close to 1.0, the transmittance retention yield of the silicone gel prepared using Si-Vi terminated phenyl T-shaped polysiloxane as the matrix decreased less with the extension of the UV irradiation time. Nevertheless, increasing the phenyl content in the silicone gel could not delay the change in the silicone gel transmittance with irradiation time.

## 4. Conclusions

Si-H-terminated T-shaped PDMS, Si-Vi-terminated T-shaped PDMS, and Ph_2_SiO unit-bridged Si-H or Si-Vi terminated linear PDMS with different polymerization degrees were meticulously prepared by equilibrium polymerization or non-equilibrium polymerization reactions to prepare silicone gels. The introduction of the Ph_2_SiO unit into polymer molecules increased the refractive index of the polymer. It made the refractive index match the crosslinking agent and the matrix to obtain optical packaging materials with high transmittance. In using Si-Vi-terminated linear PDMS or PDMS bridged with the Ph_2_SiO unit as the matrix, Si-H-terminated T-shaped PDMS prepared by non-equilibrium polymerization had higher reactivity than that obtained by equilibrium polymerization in the preparation of silicone gel. The prepared silicone gel was denser, had a higher gelation yield, and exhibited better thermal performance in a nitrogen atmosphere.

In silicone gel, when the molar ratio of SiH/SiVi was not close to the stoichiometric ratio, even if phenyl groups were introduced into the silicone gel, excessive Si-H or Si-Vi would cause the transmittance of the silicone gel to decrease rapidly with the extension of UV irradiation time. When SiH/SiVi was close to 1, the transmittance retention yield of the silicone gel prepared with Si-Vi-terminated phenyl T-shaped PDMS as the matrix, and Si-H-terminated linear PDMS containing the Ph_2_SiO unit as the crosslinking agent decreased with the increase in phenyl content. The silicone gel materials were prepared by controlling the SiH/SiVi molar ratio in the silicone gel to be close to 1.0 and using Si-Vi-terminated phenyl T-shaped polysiloxane as the matrix had better radiation resistance.

## Figures and Tables

**Table 1:** Structures of oligomers used in preparation of silicone gel.

Crosslinking Agent	Structure	Matrix	Structure
UC-616-79	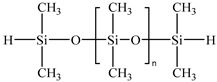	UC-273-100	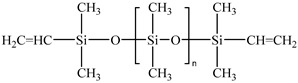
PhT-SiH	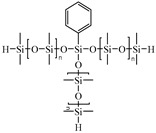	PhT-SiVi	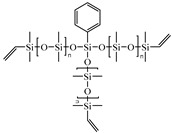
MeT-SiH	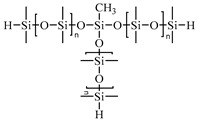	MeT-SiVi	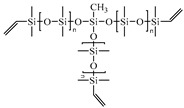
Ph_2_SiO-SiH	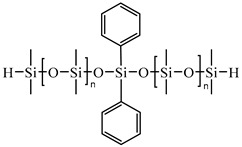	Ph_2_SiO-SiVi	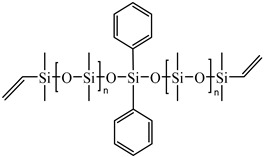

**Table 2:** Composition and gelation yield of silicone gel prepared using Si-Vi-terminated linear PDMS as matrix.

Entry	Crosslinking Agent	*M*n ^a^/(g·mol^−1^)	Matrix	*M*n ^b^	*m* ^a^/g	*m* ^b^/g	*m* ^c^/g	*m* ^d^/g	*w*_Ph_ ^e^/wt%	*w*_Ph_ ^f^/wt%	*w*_Ph_ ^g^/wt%	*r* ^h^_(Si-H)/(Si-Vi)_	Gy ^i^/%
1A	PhT-SiH-A6	2300	UC-273-100	6000	0.579	2.000	0.013	2.58 × 10^−1^	3.30	0	0.74	3.0	90.2
1B	PhT-SiH-A10	3600	UC-273-100	6000	1.000	1.932	0.015	2.93 × 10^−1^	1.98	0	0.68	3.6	92.4
1C	PhT-SiH-B6	1100	UC-273-100	6000	0.652	1.800	0.012	2.39 × 10^−1^	3.30	0	0.88	3.0	99.3
1D	PhT-SiH-B15	3000	UC-273-100	6000	1.100	1.701	0.014	2.80 × 10^−1^	2.10	0	0.82	4.2	81.3
1E	PhT-SiH-B24	4800	UC-273-100	6000	1.457	1.700	0.016	3.16 × 10^−1^	1.10	0	0.51	3.0	81.9
1F	PhT-SiH-A6	2300	Ph_2_SiO-SiVi-B6	1500	1.089	1.001	0.011	2.09 × 10^−1^	3.30	10.82	6.90	3.0	85.7
1G	PhT-SiH-B6	1100	Ph_2_SiO-SiVi-B6	1500	1.158	1.000	0.011	2.16 × 10^−1^	3.30	10.82	6.78	2.7	98.8
1H	PhT-SiH-B15	3000	Ph_2_SiO-SiVi-B18	3300	1.658	2.000	0.018	3.66 × 10^−1^	2.10	4.06	3.17	3.6	98.5
1I	PhT-SiH-B24	4800	Ph_2_SiO-SiVi-B27	5300	1.845	2.000	0.019	3.85 × 10^−1^	1.10	3.00	2.09	3.0	77.8

Note: a. crosslinking agent; b. matrix; c. Karstedt’s catalyst with a Pt concentration of 0.1 wt%; d. ECH with 0.1 wt% content in toluene solution; e. mass fraction of phenyl in the crosslinking agent; f. mass fraction of phenyl in the matrix; g. phenyl content in silicone gel; h. molar ratio of Si-H to Si-Vi in the mixture, calculated with the ^1^H NMR of the mixture before curing; i. gelation yield.

**Table 3:** Composition and gelation yield of silicone gel prepared using Si-Vi-terminated T-shaped siloxane oligomers as the matrix.

Entry	Crosslinking Agent	*M*n ^a^/(g·mol^−1^)	Matrix	*M*n ^b^	*m* ^a^/g	*m* ^b^/g	*m* ^c^/g	*m* ^d^/g	*w*_Ph_ ^e^/wt%	*w*_Ph_ ^f^/wt%	*w*_Ph_ ^g^/wt%	*r* ^h^_(Si-H)/(Si-Vi)_	Gy ^i^/%
2A	UC-616-79	7200	PhT-SiVi-B6	1500	1.001	0.200	0.006	1.20 × 10^−1^	0	3.92	0.65	0.9	82.1
2B	UC-616-79	7200	PhT-SiVi-B15	3300	1.928	1.000	0.015	2.92 × 10^−1^	0	1.66	0.57	2.4	87.9
2C	UC-616-79	7200	PhT-SiVi-B24	5400	1.896	1.150	0.015	3.05 × 10^−1^	0	1.16	0.44	3.0	83.5
2D	Ph_2_SiO-SiH-A6	3500	PhT-SiVi-B6	1500	1.485	1.000	0.013	2.49 × 10^−1^	4.62	3.92	4.34	0.9	94.1
2E	Ph_2_SiO-SiH-A12	6000	PhT-SiVi-B15	3300	0.785	1.000	0.009	1.79 × 10^−1^	2.56	1.66	2.06	1.2	91.5
2F	Ph_2_SiO-SiH-B6	1200	PhT-SiVi-B24	5400	1.210	1.000	0.011	2.21 × 10^−1^	4.24	1.16	2.85	1.2	99.6
2G	Ph_2_SiO-SiH-B18	2500	PhT-SiVi-B6	1500	1.158	1.000	0.011	2.16 × 10^−1^	2.56	3.92	3.19	0.9	91.3

Note: a. crosslinking agent; b. matrix; c. Karstedt’s catalyst with a Pt concentration of 0.1 wt%; d. ECH with 0.1 wt% content in toluene solution; e. mass fraction of phenyl in the crosslinking agent; f. mass fraction of phenyl in the matrix; g. phenyl content in silicone gel; h. molar ratio of Si-H to Si-Vi in the mixture, calculated with the ^1^H NMR of the mixture before curing; i. gelation yield.

**Table 4:** Thermal properties of silicone gels prepared using Si-Vi-terminated linear PDMS as matrix.

Entry	*T* ^a^_5%_/°C	*T* ^b^_10%_/°C	*T* ^c^_20%_/°C	*T* ^d^_50%_/°C	*WR* ^e^/wt%	First Thermal Decomposition		Second Thermal Decomposition	
						MTDT ^f^/°C	TDR ^g^/(wt%.°C^−1^)	MTDT ^f^/°C	TDR ^g^/(wt%.°C^−1^)
1A	437.2	460.6	489.2	530.8	1.45	543.7	−1.08	-	-
1B	403.3	431.0	471.7	538.2	10.54	529.1	−0.64	586.3	-0.53
1C	354.6	440.5	492.3	569.6	20.76	534.3	−0.42	566.5	−0.40
1D	406.3	442.6	482.3	558.3	10.14	511.3	−0.45	611.3	−0.58
1E	460.5	497.7	541.2	623.9	6.07	641.6	−1.15	-	-
1F	412.3	445.6	485.3	577.0	30.85	525.4	−0.38	583.4	−0.44
1G	358.8	406.7	461.8	536.5	3.67	507.1	−0.59	560.9	−1.01
1H	434.2	465.2	499.6	567.9	6.43	591.6	−0.79	-	-
1I	403.0	436.6	479.1	593.0	18.51	488.9	−0.27	602.7	−0.82

Note: a. temperature at 5% weight loss; b. temperature at 10% weight loss; c. temperature at 20% weight loss; d. temperature at 50% weight loss; e. residual weight at 800 °C; f. maximum thermal decomposition temperature; g. thermal decomposition rate at the maximum thermal decomposition temperature.

**Table 5:** Thermal properties of silicone gels prepared using Si-Vi-terminated T-shaped siloxane oligomers as the matrix.

Entry	*T* ^a^_5%_/°C	*T* ^b^_10%_/°C	*T* ^c^_20%_/°C	*T* ^d^_50%_/°C	*WR* ^e^/wt%	First Thermal Decomposition		Second Thermal Decomposition	
						MTDT ^f^/°C	TDR ^g^/(wt%.°C^−1^)	MTDT ^f^/°C	TDR ^g^/(wt%.°C^−1^)
2A	441.4	471.8	504.3	575.3	19.15	539.4	−0.44	576.0	−0.44
2B	397.4	443.7	484.2	550.8	16.95	533.2	−0.53	587.4	−0.60
2C	345.3	411.4	468.2	533.6	19.83	531.1	−0.65	-	-
2D	421.2	458.4	490.4	549.4	2.39	543.8	−0.56	589.6	−0.81
2E	397.5	441.4	484.2	550.5	17.00	533.6	−0.54	586.5	−0.59
2F	437.5	480.0	528.2	613.6	11.07	632.2	−1.12	-	-
2G	233.8	383.8	461.4	543.4	9.26	530.5	−0.50	579.4	−0.49

Note: a. temperature at 5% weight loss; b. temperature at 10% weight loss; c. temperature at 20% weight loss; d. temperature at 50% weight loss; e. residual weight at 800 °C; f. maximum thermal decomposition temperature; g. thermal decomposition rate at the maximum thermal decomposition temperature.

**Table 6:** Silicone gels prepared by hydrosilylation reaction with crosslinking agents and matrix of different structures.

Entry	Crosslinking Agent ^a^	*M*n ^b^/(g·mol^−1^)	Matrix ^c^	*M*n ^d^/(g·mol^−1^)	*m* ^e^/g	*m* ^f^/g	*m* ^g^/g	*M* ^h^/g	*w*_Ph_ ^i^/wt%	*r* ^j^_(Si-H)/(Si-Vi)_
3A	PhT-SiH-A4	1700	UC-273-100	6000	0.584	2.500	0.016	3.09 × 10^−1^	0.88	3.75
3B	PhT-SiH-A6	2300	UC-273-100	6000	0.628	2.360	0.015	2.99 × 10^−1^	0.69	3.06
3C	PhT-SiH-A10	3600	UC-273-100	6000	1.035	2.000	0.015	3.04 × 10^−1^	0.67	3.56
3D	PhT-SiH-A20	5100	UC-273-100	6000	1.243	1.800	0.015	3.04 × 10^−1^	0.60	3.54
3E	MeT-SiH-A33 ^k^	4500	UC-273-100	6000	1.530	1.800	0.017	3.34 × 10^−1^	0.0	2.90
3F	UC-616-79	7200	PhT-SiVi-B6	1500	3.003	0.600	0.018	3.60 × 10^−1^	0.65	0.91
3G	UC-616-79	7200	PhT-SiVi-B15	3300	2.005	1.040	0.015	3.05 × 10^−1^	0.57	0.82
3H	UC-616-79	7200	PhT-SiVi-B24	5400	1.978	1.200	0.016	3.18 × 10^−1^	0.44	1.01
3I	UC-616-79	7200	MeT-SiVi-B26 ^l^	6100	2.105	1.200	0.017	3.30 × 10^−1^	0.0	1.20

Note: a. crosslinking agent; b. number-average molecular weight of synthesized PhT-SiH (Entry 3A~3E) or UC-616-79; c. matrix; d. number-average molecular weight of UC-273-100 or synthesized PhT-SiVi (Entry 3F~3I); e. mass of crosslinking agent; f. mass of matrix; g. Karstedt’s catalyst (Pt content is 0.1 wt%); h. ECH with 0.1 wt% content in toluene solution; i. phenyl content in the sample, *w*_Ph_ is the mass fraction of phenyl in the cured product; j. *r*(Si-H)/(Si-Vi) is the molar ratio of Si-H to Si-Vi in the mixed material, calculated with the ^1^H NMR of the mixture before curing. k. In Scheme 1b, the T-shaped Si-H containing crosslinking agent prepared by replacing PhSiCl_3_ with MeSiCl_3_. l. In Scheme 3, the T-shaped Si-Vi containing matrix prepared by replacing PhSiCl_3_ with MeSiCl_3_.

**Table 7:** Effect of the SiH/SiVi ratio and phenyl content on the transmittance of silicone gel containing a Ph_2_SiO unit.

Entry	Crosslinking Agent	*M*n ^a^/(g·mol^−1^)	Matrix	*M*n ^b^/(g·mol^−1^)	*m* ^c^/g	*m* ^d^/g	*m* ^e^/g	*m* ^f^/g	*w*_Ph_ ^g^/wt%	*r* ^h^_(Si-H)/(Si-Vi)_
4A	PhT-SiH-A4	1700	Ph_2_SiO-SiVi-B6	1400	0.584	2.500	0.016	3.09 × 10^−1^	9.65	0.30
4B	PhT-SiH-A6	2300	Ph_2_SiO-SiVi-B6	1400	0.628	2.360	0.015	2.99 × 10^−1^	9.24	0.24
4C	PhT-SiH-A10	3600	Ph_2_SiO-SiVi-B6	1400	1.035	2.000	0.015	3.04 × 10^−1^	7.81	0.28
4D	PhT-SiH-A20	5100	Ph_2_SiO-SiVi-B6	1400	1.530	1.800	0.017	3.34 × 10^−1^	6.53	0.35
4E	Ph_2_SiO-SiH-B6	1800	PhT-SiVi-B6	1500	1.931	1.3	0.016	3.23 × 10^−1^	6.65	1.07
4F	Ph_2_SiO-SiH-B18	3000	PhT-SiVi-B6	1500	2.09	1	0.016	3.09 × 10^−1^	4.74	0.91
4G	Ph_2_SiO-SiH-B27	4200	PhT-SiVi-B6	1500	2.511	0.8	0.017	3.31 × 10^−1^	3.73	0.98

Note: a. molecular weight of the crosslinking agent; b. molecular weight of the matrix; c. mass of the crosslinking agent; d. mass of the matrix; e. Karstedt catalyst (Pt content is 0.1 wt%); f. ECH with 0.1 wt% content in toluene solution; g. mass fraction of phenyl in the cured product; h. molar ratio of Si-H to Si-Vi in the mixture, calculated from the ^1^H NMR of the mixture before curing.

## Data Availability

Structure characterizations of Ph_2_SiO-bridged Si-H-terminated linear siloxane oligomers, Si-Vi-terminated T-branched siloxane oligomers, and Ph_2_SiO-bridged Si-Vi-terminated linear siloxane oligomers can be found in the Supplementary Information of this manuscript.

## Supplementary Materials

The following supporting information can be downloaded at: https://www.mdpi.com/article/10.3390/polym17010009/s1, Table S1: Preparation of Si-H terminated T-branched siloxane oligomers (PhT-SiH-A) by cationic catalyzed ring-opening polymerization reaction; Table S2: Preparation of Si-H terminated T-branched siloxane oligomers (PhT-SiH-B) by “living” anionic ring-opening polymerization reaction; Table S3: Preparation of Ph_2_SiO bridged Si-H terminated linear siloxane oligomers (Ph_2_SiO-SiH-A) by a cationic ring-opening polymerization method; Table S4: Preparation of Ph_2_SiO bridged Si-H terminated linear siloxane oligomers (Ph_2_SiO-SiH-B) by “living” anionic ring-opening polymerization reaction; Table S5: Preparation of Si-Vi terminated T-branched siloxane oligomers (PhT-SiVi-B) by “living” anionic ring-opening polymerization reaction; Table S6: Preparation of Ph_2_SiO bridged Si-Vi terminated linear siloxane oligomers (Ph_2_SiO-SiVi-B) by “living” anionic ring-opening polymerization reaction; Figure S1: The ^1^H NMR spectrum (a), ^13^C NMR spectrum (b), ^29^Si NMR spectrum (c), FT-IR spectrum (d), and GPC curve (e) of Si-H terminated T-shaped siloxane oligomer sample (Table S2, Entry PhT-SiH-B6), CDCl_3_ was used as the solvent in NMR analysis; Figure S2: The ^1^H NMR spectrum (a), ^13^C NMR spectrum (b), ^29^Si NMR spectrum (c), FT-IR spectrum (d), and GPC curve (e) of Ph_2_SiO bridged Si-H terminated linear siloxane oligomer sample (Table S4, Entry Ph_2_SiO-SiH-B18), CDCl_3_ was used as the solvent in NMR analysis; Figure S3: The ^1^H NMR spectrum (a), ^13^C NMR spectrum (b), ^29^Si NMR spectrum (c), FT-IR spectrum (d), and GPC curve (e) of Si-Vi terminated T-shaped siloxane oligomer sample (Table S5, Entry PhT-SiVi-B15), CDCl_3_ was used as the solvent in NMR analysis; Figure S4: The ^1^H NMR spectrum (a), ^13^C NMR spectrum (b), FT-IR spectrum (c), and GPC curve (d) of Ph_2_SiO bridged Si-Vi terminated linear siloxane oligomers (Ph_2_SiO-SiVi) (Table S6, Entry Ph_2_SiO-SiVi-B18), CDCl_3_ was used as the solvent in NMR analysis. The structural analysis of each kind of polymers is provided in Section III of the Supplementary Materials.
