# Supplementary material for: Preparation, Optical, and Heat Resistance Properties of Phenyl-Modified Silicone Gel
Source: Polymers (Basel). 2024 Dec 24;17(1):9. doi: 10.3390/polym17010009 (PMC11722829; doi:10.3390/polym17010009)
Supplement: Supplementary file 1 [file polymers-17-00009-s001.zip › polymers-3318455-supplementary.pdf]

Supporting Information

Part I. The amount of various raw materials used to synthesize each polymer, the reaction yield, and analysis results of each polymer.

Table S1. Preparation of Si-H terminated T-branched siloxane oligomers (PhT-SiH-A) by cationic catalyzed ring-opening polymerization reaction

Entry	n_{desn}[a]	Mn_{desn}[b]/($g\cdot mol^{-1}$)	m[c]/g	m[d]/g	m[e]/g	n_{exptl}	Mn/($g\cdot mol^{-1}$)	Mw/($g\cdot mol^{-1}$)	PDI	η_{25}/cP	n_D^{25}	Yield/%
PhT-SiH-A4	4	1220	49.61	133.35	5.49	6	1800	7400	4.11	26.00	1.4175	74.6
PhT-SiH-A6	6	1660	33.07	133.48	5.00	9	2300	6600	2.87	29.50	1.4139	85.9
PhT-SiH-A10	10	2550	22.33	152.23	5.56	16	3600	16000	4.44	60.25	1.4103	88.6
PhT-SiH-A20	20	4780	11.02	148.31	4.78	22	4300	28800	6.70	145.10	1.4081	67.5

Note. a. n_{desn} is the designed degree of polymerization of the polymer; b. Mn_{desn} is the designed molecular weight of the polymer; c. PTDS; d. D_4; e. HND-580.

Table S2. Preparation of Si-H terminated T-branched siloxane oligomers (PhT-SiH-B) by "living" anionic ring-opening polymerization reaction

Entry	n_{desn}[a]	Mn_{desn}[b]/($g\cdot mol^{-1}$)	V[c]/mL	m[d]/g	m[e]/g	n_{exptl}	Mn/($g\cdot mol^{-1}$)	Mw/($g\cdot mol^{-1}$)	PDI	η_{25}/cP	n_D^{25}	Yield/%
PhT-SiH-B6	6	1660	270.0	120.00	20.92	9	1100	2100	1.91	11.75	1.4150	81.6
PhT-SiH-B15	15	3670	107.9	120.00	8.53	15	3000	8100	2.70	37.00	1.4080	93.6
PhT-SiH-B24	24	5670	67.4	120.00	5.20	30	4800	12700	2.65	60.75	1.4062	95.7

Note. a. n_{desn} is the designed degree of polymerization of the polymer; b. Mn_{desn} is the designed molecular weight of the polymer; c. the volume of 1 mol/L lithium dimethylsilanol solution; d. D_3; e. the mass of $PhSiCl_3$ (1.1 times the molar number of lithium dimethylsilanol).

Table S3. Preparation of Ph_2SiO bridged Si-H terminated linear siloxane oligomers (Ph_2SiO-SiH-A) by a cationic ring-opening polymerization method

Entry	$n_{desn}{}^{a}$	$Mn_{desn}{}^{b}$/(g·mol^{-1})	m^{c}/g	m^{d}/g	m^{e}/g	n_{exptl}	Mn/(g·mol^{-1})	Mw/(g·mol^{-1})	PDI	η_{25}/cP	$n_D{}^{25}$	Yield/%
Ph_2SiO-SiH-A6	6	1220	49.84	133.48	5.50	9	3500	6300	1.80	26.25	1.4322	85.9
Ph_2SiO-SiH-A12	12	2110	27.69	148.31	5.28	18	6000	13200	2.20	51.75	1.4198	87.4

Note. a. n_{desn} is the designed degree of polymerization of the polymer; b. Mn$_{desn}$ is the designed molecular weight of the polymer; c. TMDPTS; d. D_4; e. HND-580.

Table S4. Preparation of Ph_2SiO bridged Si-H terminated linear siloxane oligomers (Ph_2SiO-SiH-B) by "living" anionic ring-opening polymerization reaction

Entry	$n_{desn}{}^{a}$	$Mn_{desn}{}^{b}$/(g·mol^{-1})	V^{c}/mL	m^{d}/g	m^{e}/g	n_{exptl}	Mn/(g·mol^{-1})	Mw/(g·mol^{-1})	PDI	η_{25}/cP	$n_D{}^{25}$	Yield/%
Ph_2SiO-SiH-B6	6	1220	135.0	60.00	18.80	10	1200	1800	1.50	15.75	1.4375	84.8
Ph_2SiO-SiH-B18	18	3000	45.0	60.00	6.27	18	2500	4800	1.92	29.25	1.4160	95.0
Ph_2SiO-SiH-B27	27	4330	30.0	60.00	4.18	26	4200	7800	1.86	46.50	1.4120	98.9

Note. a. n_{desn} is the designed degree of polymerization of the polymer; b. Mn$_{desn}$ is the designed molecular weight of the polymer; c. the volume of 1 mol/L lithium dimethylsilanol solution; d. D_3; e. the mass of Ph_2SiCl_2 (1.1 times the molar number of lithium dimethylsilanol).

Table S5. Preparation of Si-Vi terminated T-branched siloxane oligomers (PhT-SiVi-B) by "living" anionic ring-opening polymerization reaction

Entry	$n_{desn}{}^{a}$	$Mn_{desn}{}^{b}$/(g·mol^{-1})	V^{c}/mL	$m_1{}^{d}$/g	$m_2{}^{e}$/g	n_{exptl}	Mn/(g·mol^{-1})	Mw/(g·mol^{-1})	PDI	η_{25}/cP	$n_D{}^{25}$	Yield/%
PhT-SiVi-B6	6	1740	270.0	120.00	20.92	7	1500	2400	1.60	16.75	1.4200	92.9

PhT-SiVi-B15	15	3740	107.9	120.00	8.53	19	3300	5500	1.67	33.75	1.4120	91.4
PhT-SiVi-B24	24	5740	67.4	120.00	5.20	28	5400	8600	1.59	53.00	1.4091	91.6

Note. a. n_{desn} is the designed degree of polymerization of the polymer; b. Mn_{desn} is the designed molecular weight of the polymer; c. the volume of 1 mol/L lithium dimethylvinylsilanol solution; d. D_3; e. the mass of PhSiCl3 (1.1 times the molar number of lithium dimethylvinylsilanol).

Table S6. Preparation of Ph_2SiO bridged Si-Vi terminated linear siloxane oligomers (Ph_2SiO-SiVi-B) by "living" anionic ring-opening polymerization reaction

Entry	n_{desn}[a]	Mn_{desn}[b]/(g·mol^{-1})	V[c]/mL	m_1[d]/g	m_2[e]/g	n_{exptl}	Mn/(g·mol^{-1})	Mw/(g·mol^{-1})	PDI	η_{25}/cP	n_D^{25}	Yield/%
Ph_2SiO-SiVi-B6	6	1270	135.0	60.00	18.80	7	1400	2000	1.43	16.00	1.4372	93.4
Ph_2SiO-SiVi-B18	18	3050	45.0	60.00	6.27	23	3600	5700	1.58	38.50	1.4180	94.5
Ph_2SiO-SiVi-B27	27	4390	30.0	60.00	4.18	32	5300	8100	1.53	53.25	1.4135	94.6

Note. a. n_{desn} is the designed degree of polymerization of the polymer; b. Mn_{desn} is the designed molecular weight of the polymer; c. the volume of 1 mol/L lithium dimethylvinylsilanol solution; d. D_3; e. the mass of Ph_2SiCl_2 (1.1 times the molar number of lithium dimethylvinylsilanol).

Part II. Structural characterization of polymers

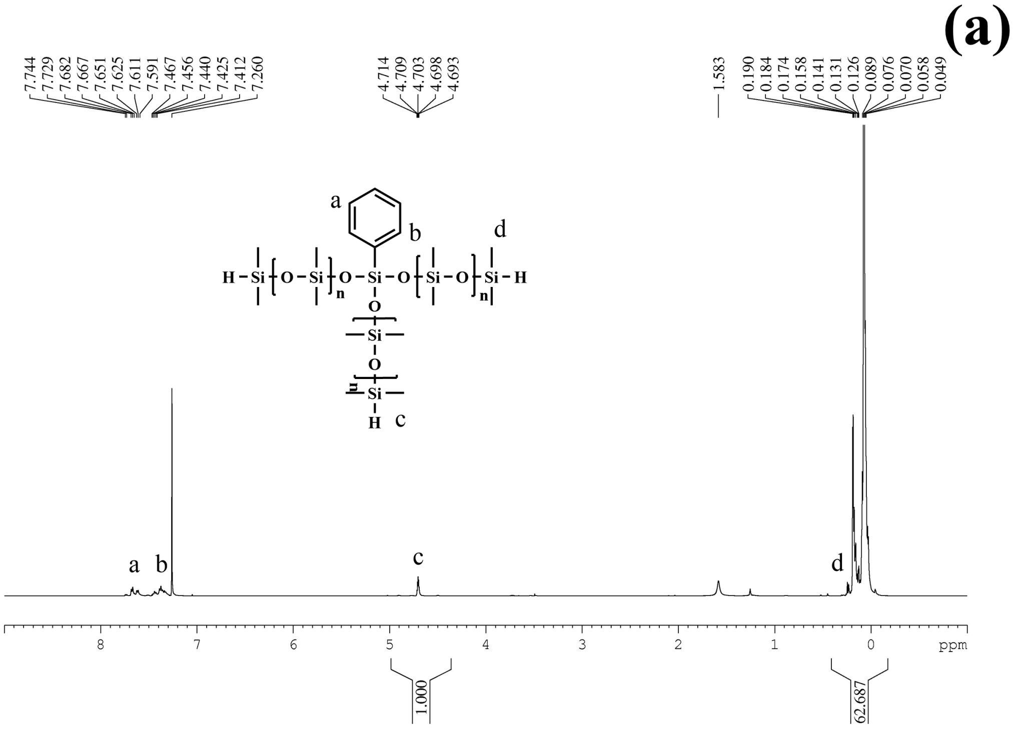

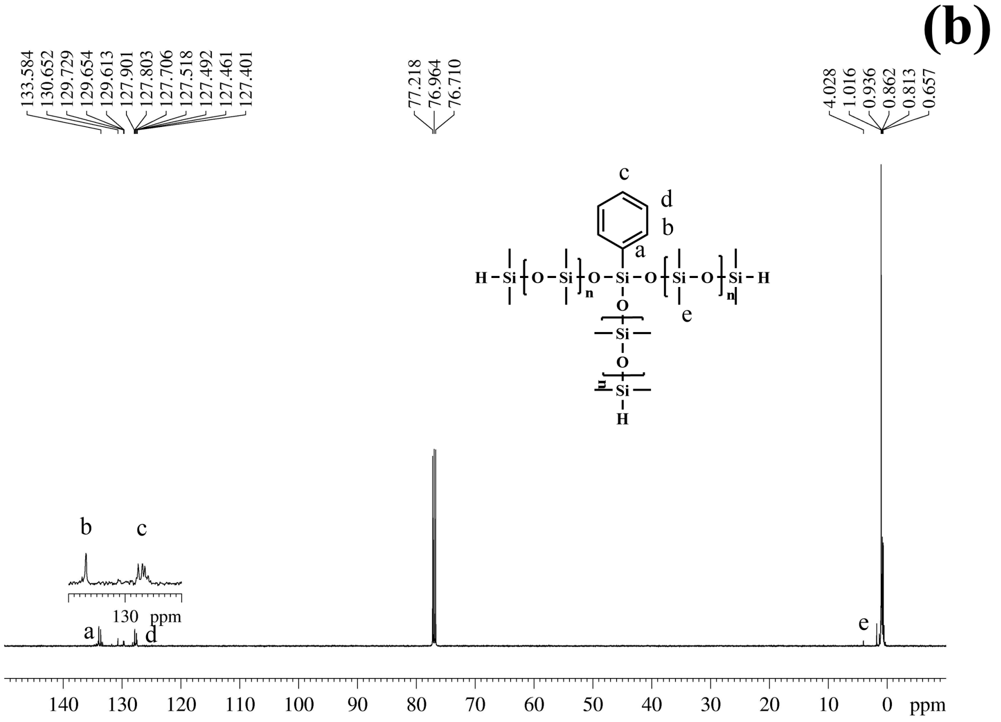

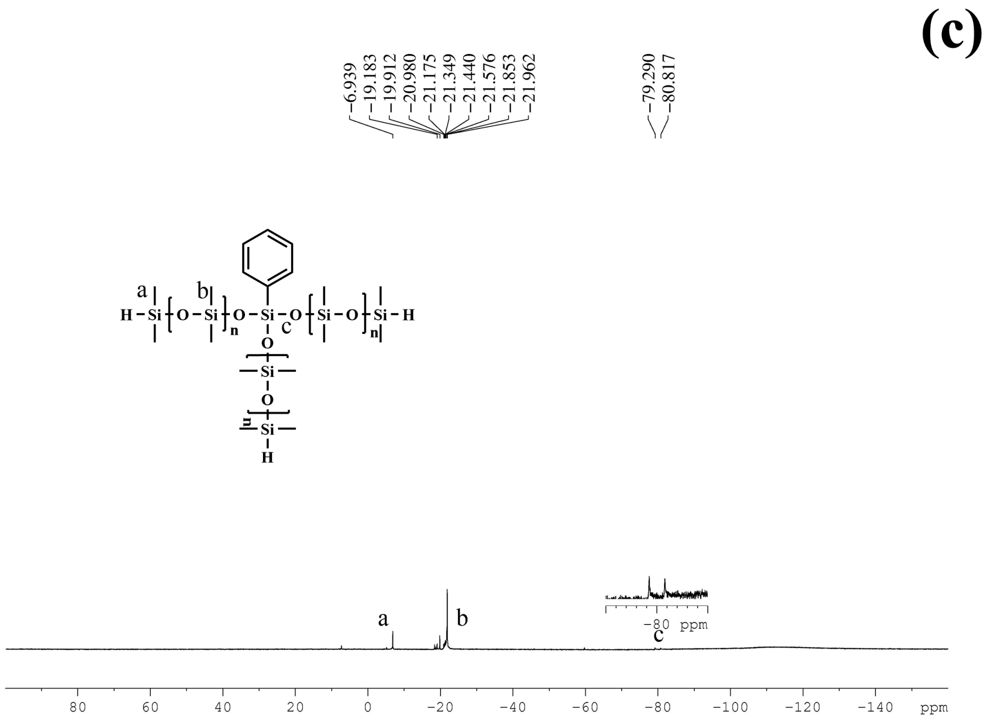
(c)
-6.939
-19.183
-19.912
-20.980
-21.175
-21.349
-21.440
-21.576
-21.853
-21.962
-79.290
-80.817
a
b
c
-80 ppm
80 60 40 20 0 -20 -40 -60 -80 -100 -120 -140 ppm

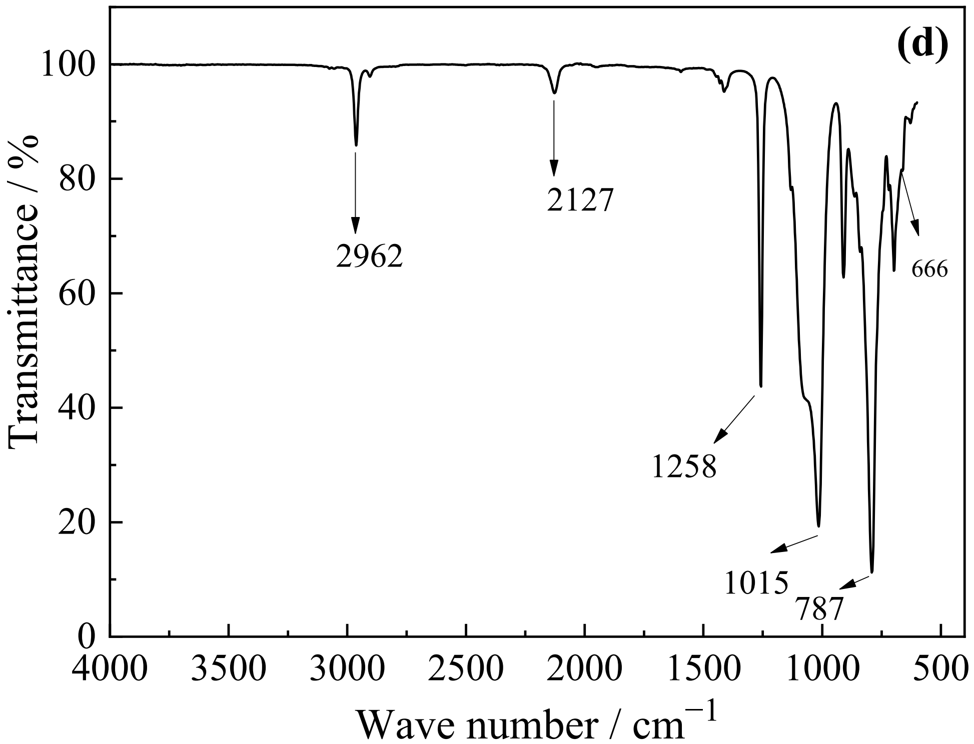
(d)
Transmittance / %
Wave number / cm^{-1}
2962
2127
1258
1015
787
666
4000 3500 3000 2500 2000 1500 1000 500
0 20 40 60 80 100

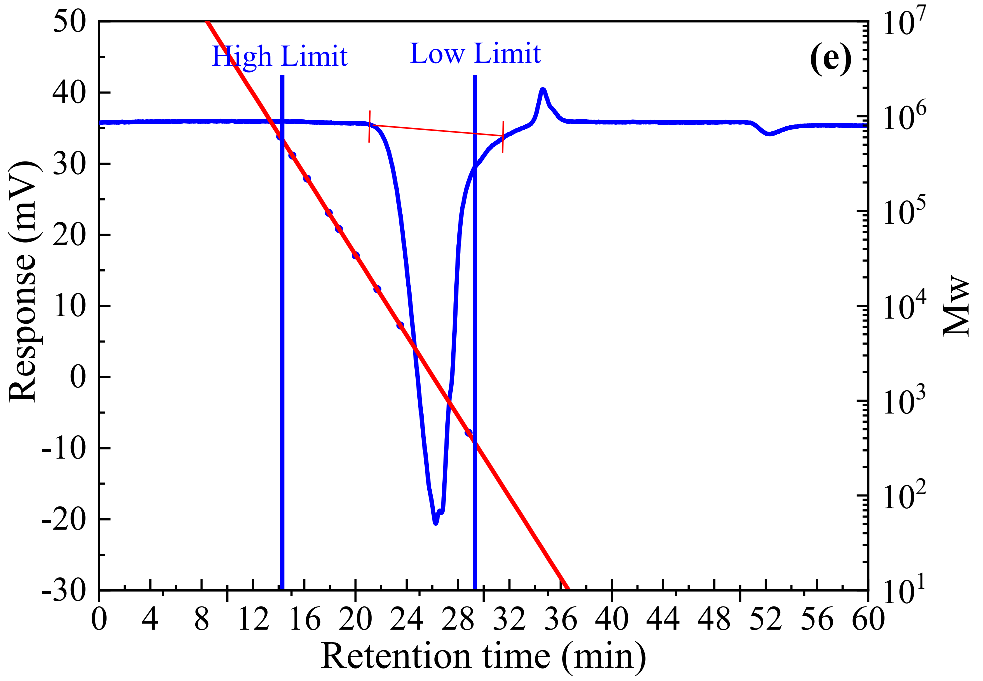

Figure S1. The ^{1}H NMR spectrum (a), ^{13}C NMR spectrum (b), ^{29}Si NMR spectrum (c), FT-IR spectrum (d), and GPC curve (e) of Si-H terminated T-shaped siloxane oligomer sample (Table S2, Entry PhT-SiH-B6), $CDCl_3$ was used as the solvent in NMR analysis.

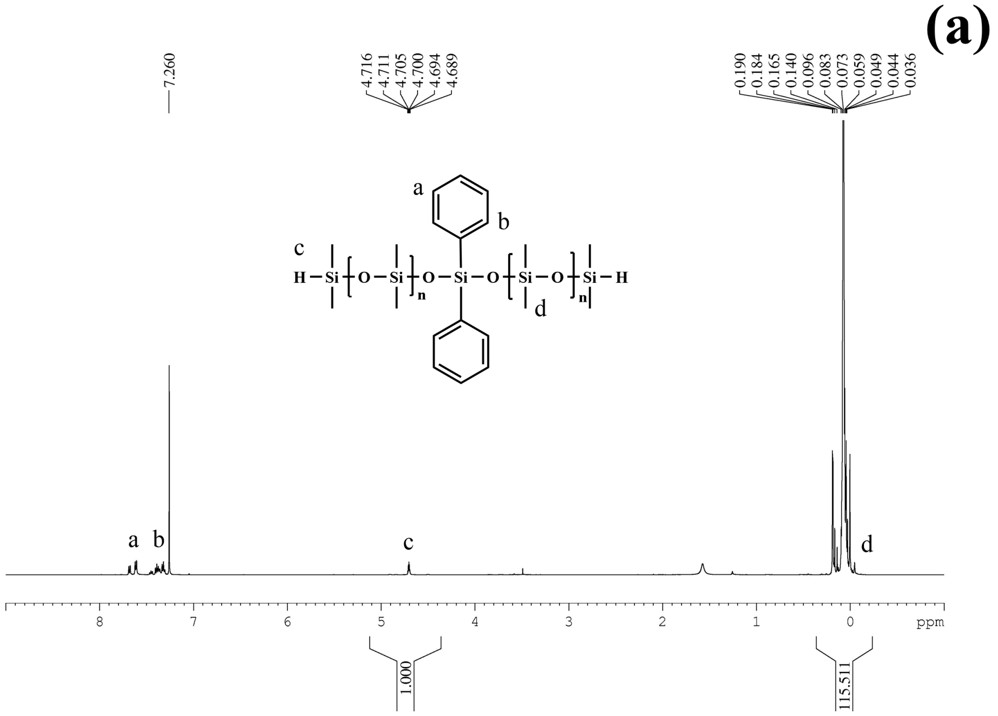

(b)

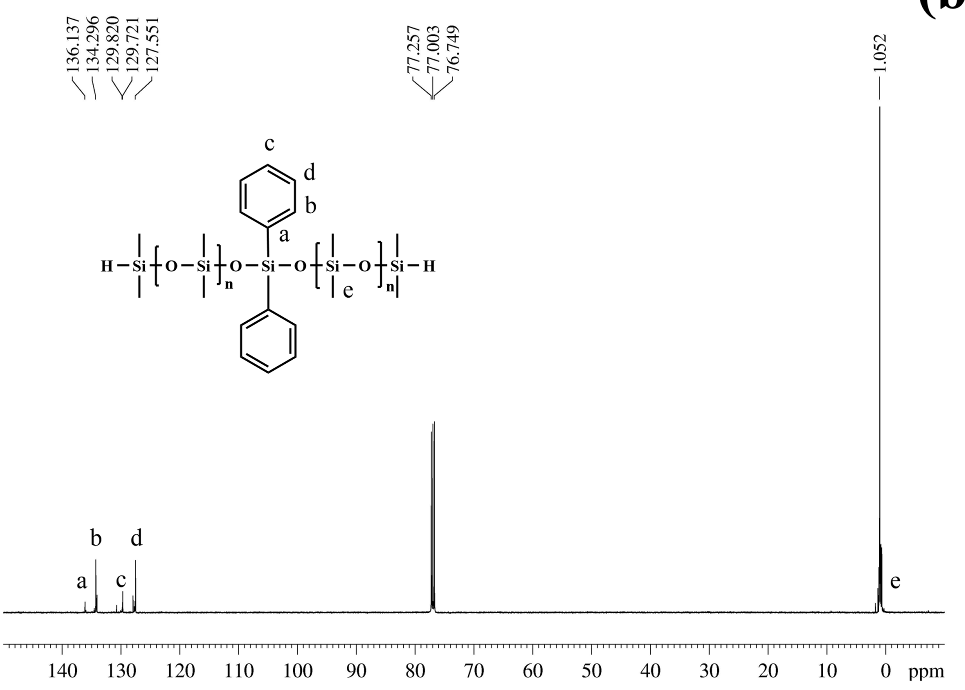
136.137
134.296
129.820
129.721
127.551
77.257
77.003
76.749
1.052
c
d
b
a
e
n
140 130 120 110 100 90 80 70 60 50 40 30 20 10 0 ppm

(c)

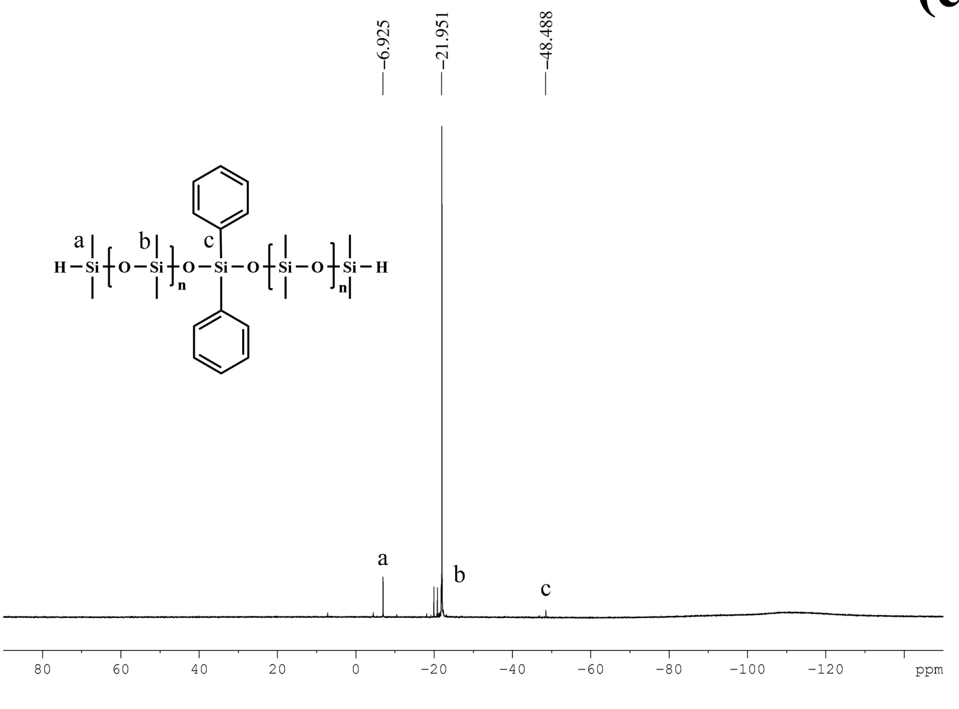
-6.925
-21.951
-48.488
a
b
c
n
80 60 40 20 0 -20 -40 -60 -80 -100 -120 ppm

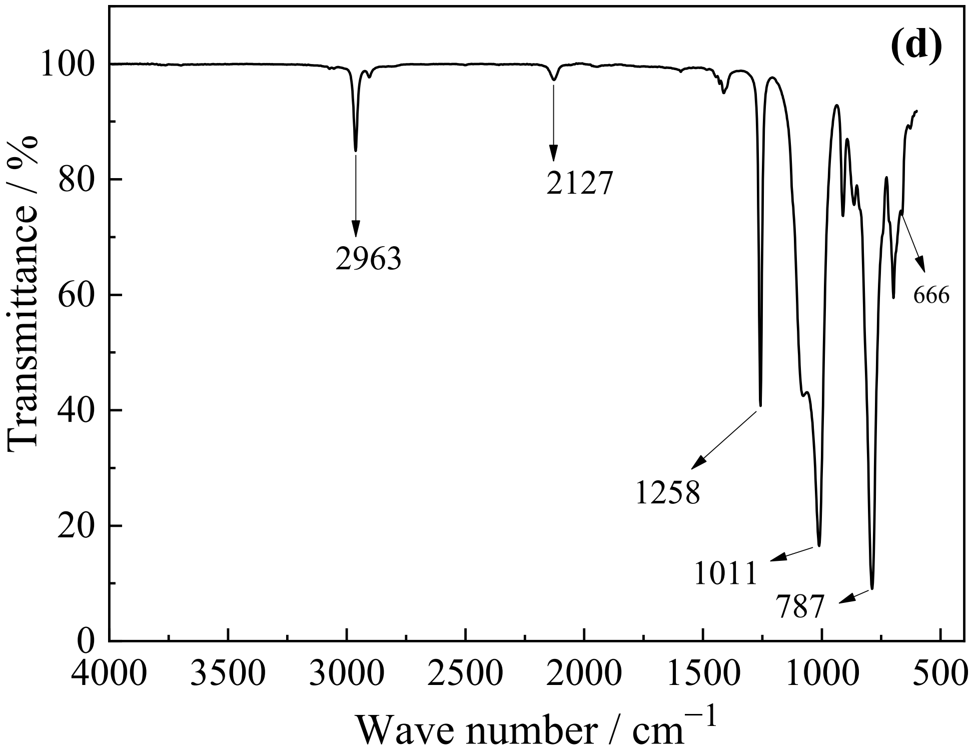

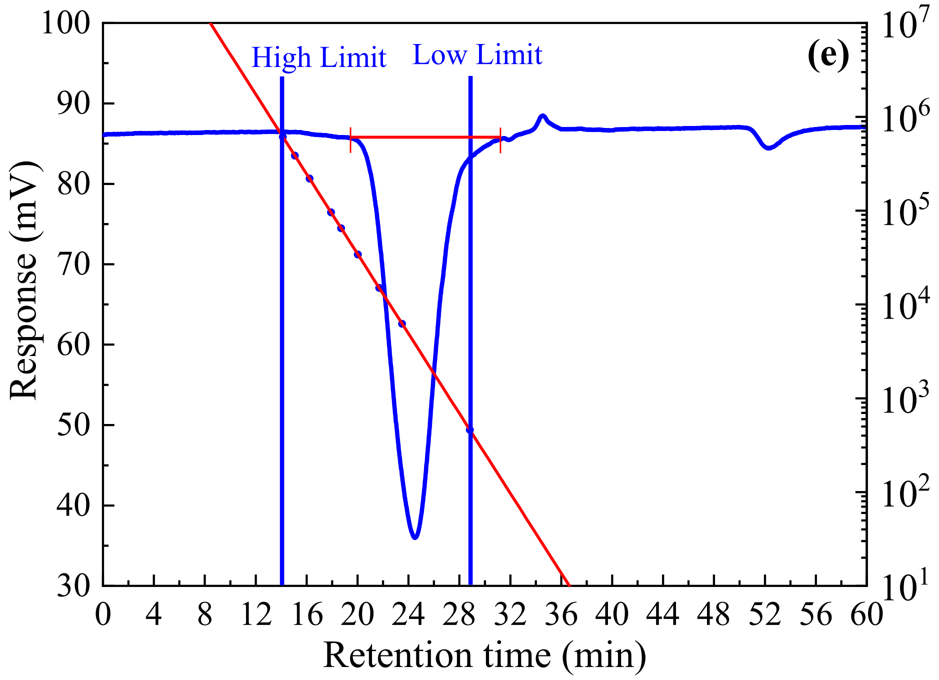

Figure S2. The ^{1}H NMR spectrum (a), ^{13}C NMR spectrum (b), ^{29}Si NMR spectrum (c), FT-IR spectrum (d), and GPC curve (e) of Ph_2SiO bridged Si-H terminated linear siloxane oligomer sample (Table S4, Entry Ph_2SiO-SiH-B18), $CDCl_3$ was used as the solvent in NMR analysis.

(a)

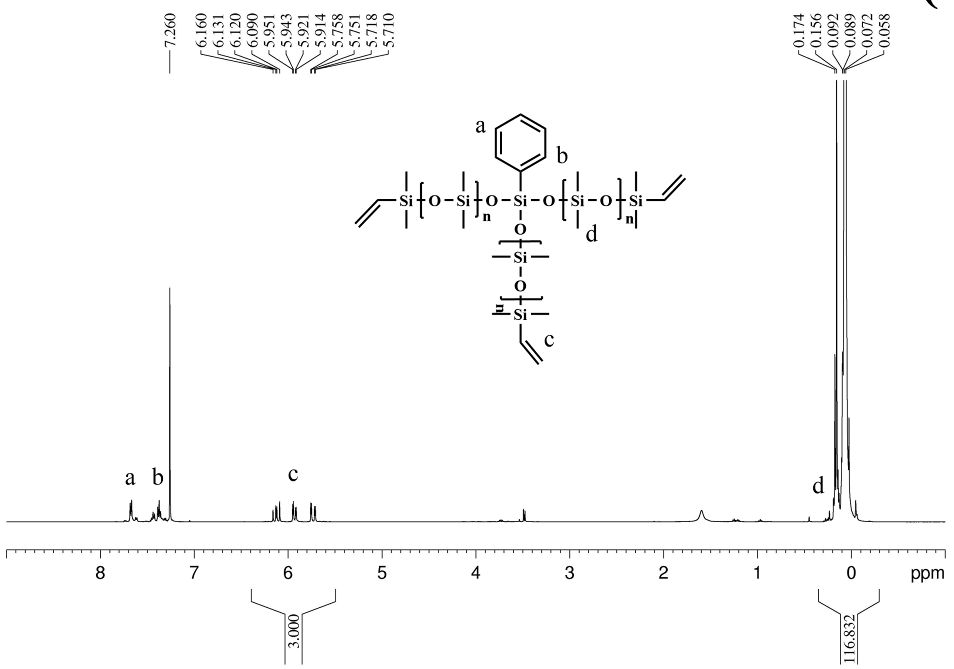
7.260
6.160
6.131
6.120
6.090
5.951
5.943
5.921
5.914
5.758
5.751
5.718
5.710
0.174
0.156
0.092
0.089
0.072
0.058
a
b
c
d
n
n
n
8
7
6
5
4
3
2
1
0
ppm
3.000
116.832

(b)

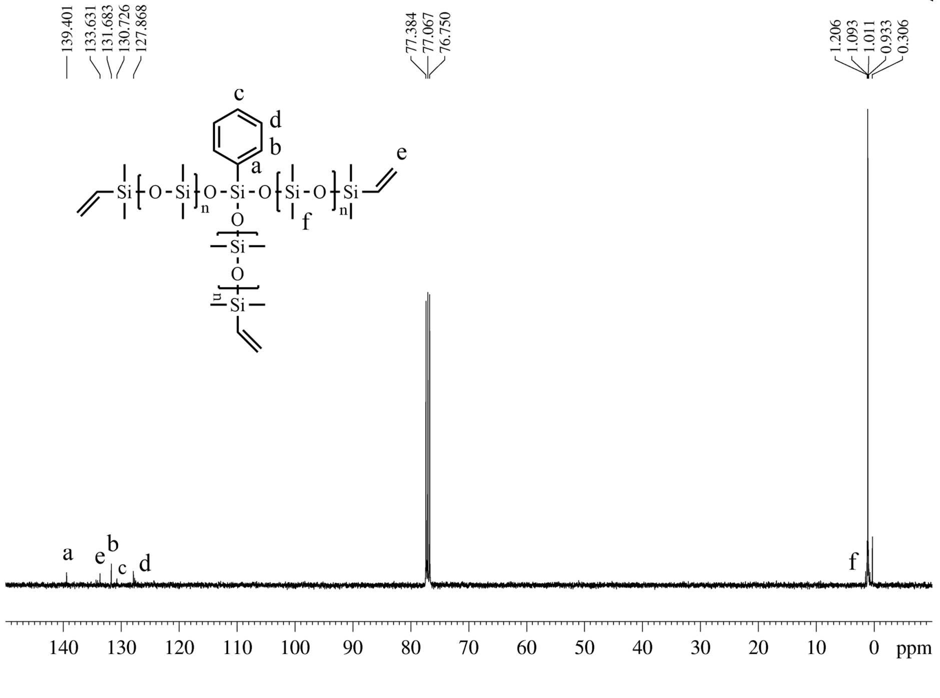
139.401
133.631
131.683
130.726
127.868
77.384
77.067
76.750
1.206
1.093
1.011
0.933
0.306
a
b
c
d
e
f
n
n
n
140
130
120
110
100
90
80
70
60
50
40
30
20
10
0
ppm

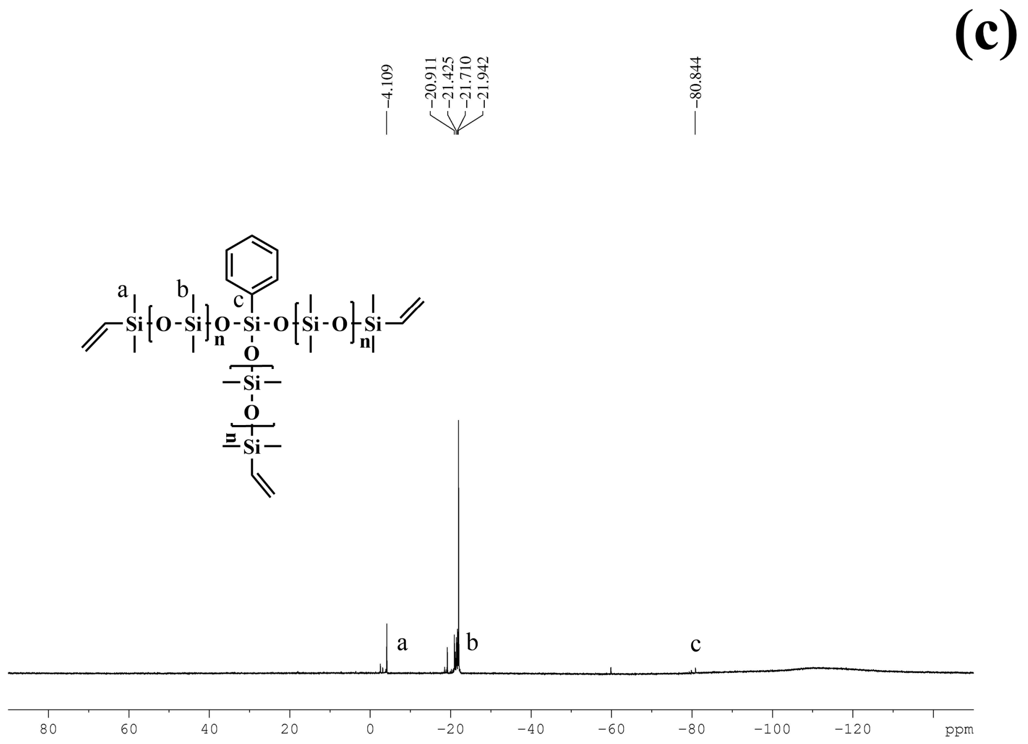
(c)
-4.109
-20.911
-21.425
-21.710
-21.942
-80.844
a
b
c
80 60 40 20 0 -20 -40 -60 -80 -100 -120 ppm

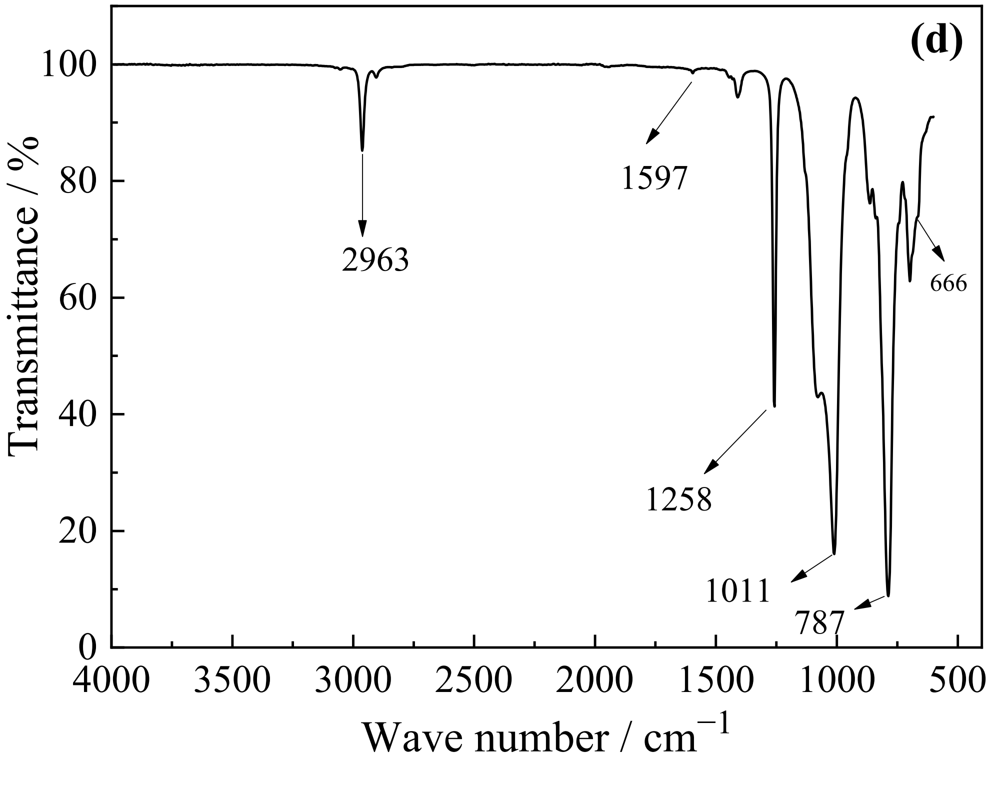
(d)
Transmittance / %
Wave number / cm^{-1}
2963
1597
1258
1011
787
666

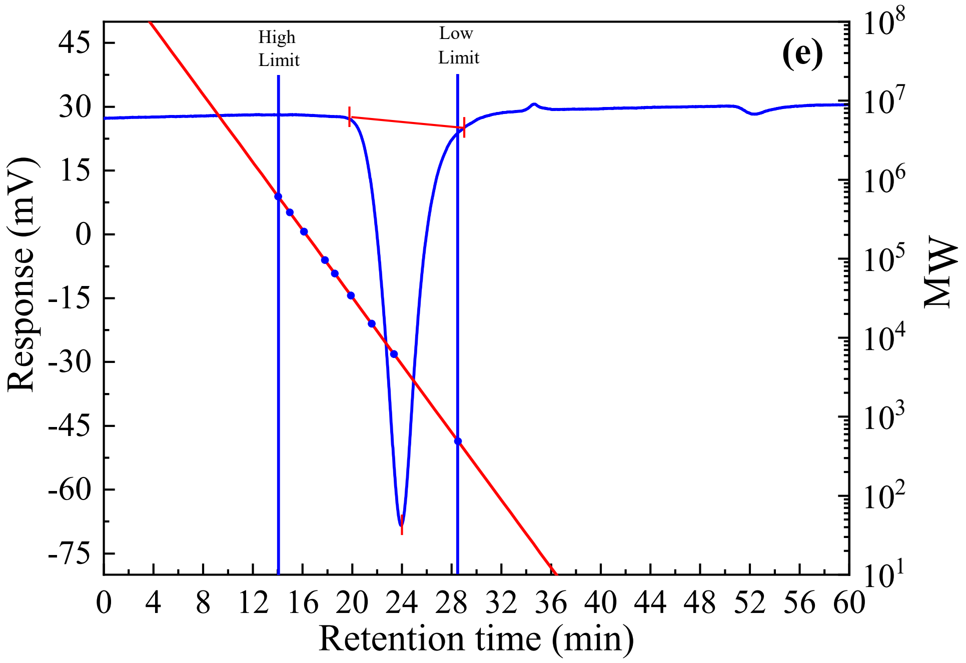

Figure S3. The ^{1}H NMR spectrum (a), ^{13}C NMR spectrum (b), ^{29}Si NMR spectrum (c), FT-IR spectrum (d), and GPC curve (e) of Si-Vi terminated T-shaped siloxane oligomer sample (Table S5, Entry PhT-SiVi-B15), $CDCl_3$ was used as the solvent in NMR analysis.

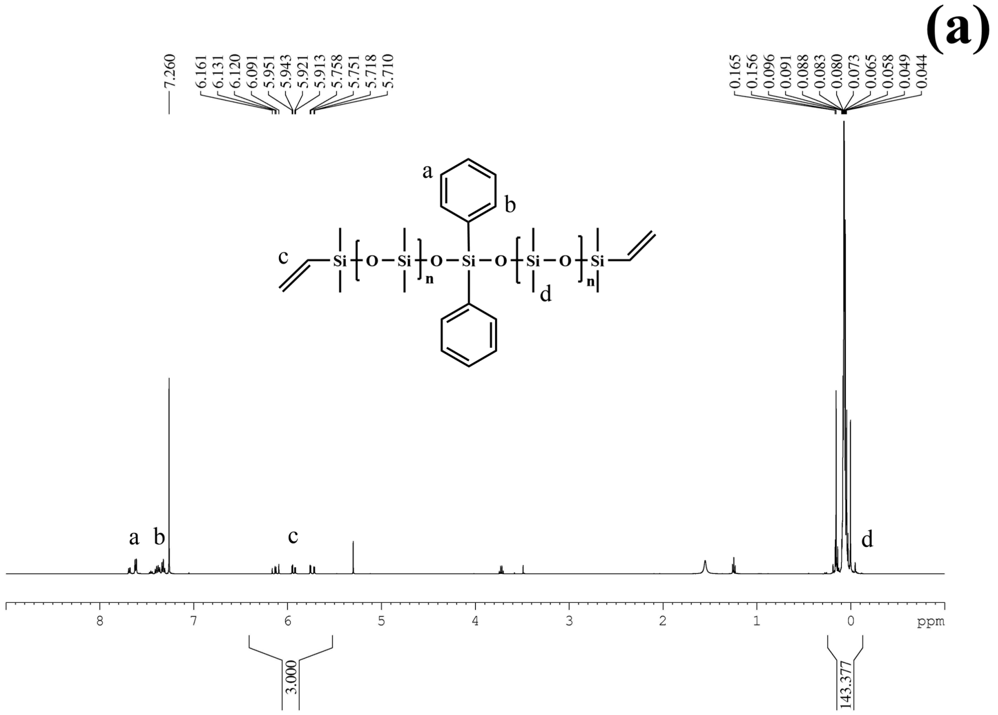

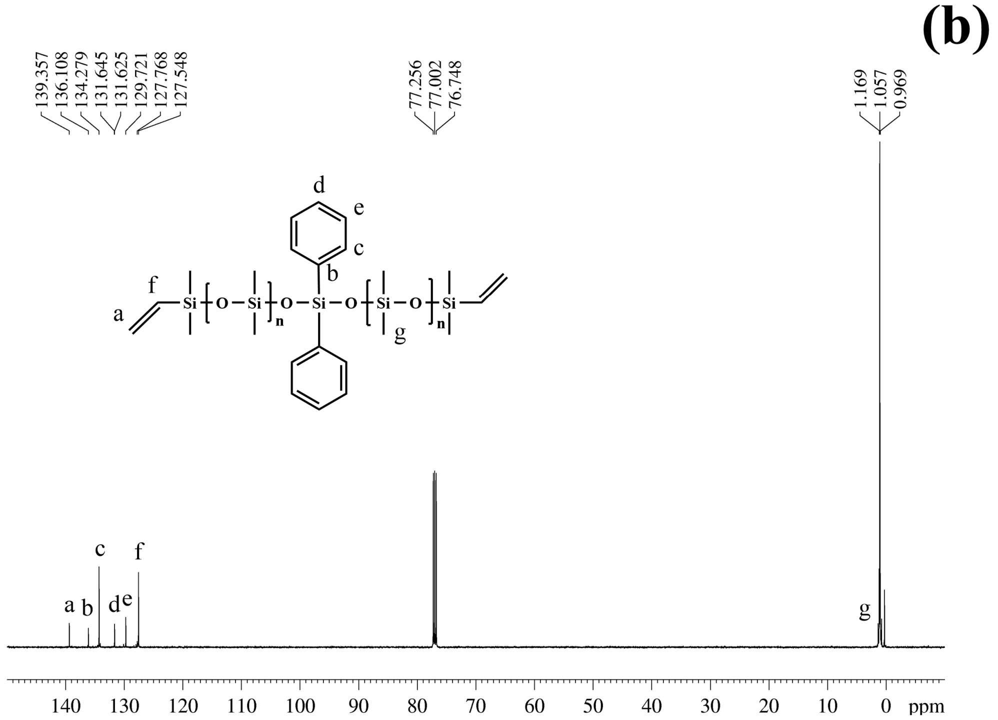
(b)
139.357
136.108
134.279
131.645
131.625
129.721
127.768
127.548
77.256
77.002
76.748
1.169
1.057
0.969
d
e
c
b
f
a
Si
O
Si
n
O
Si
O
Si
O
Si
g
n
a b c d e f
g
140 130 120 110 100 90 80 70 60 50 40 30 20 10 0 ppm

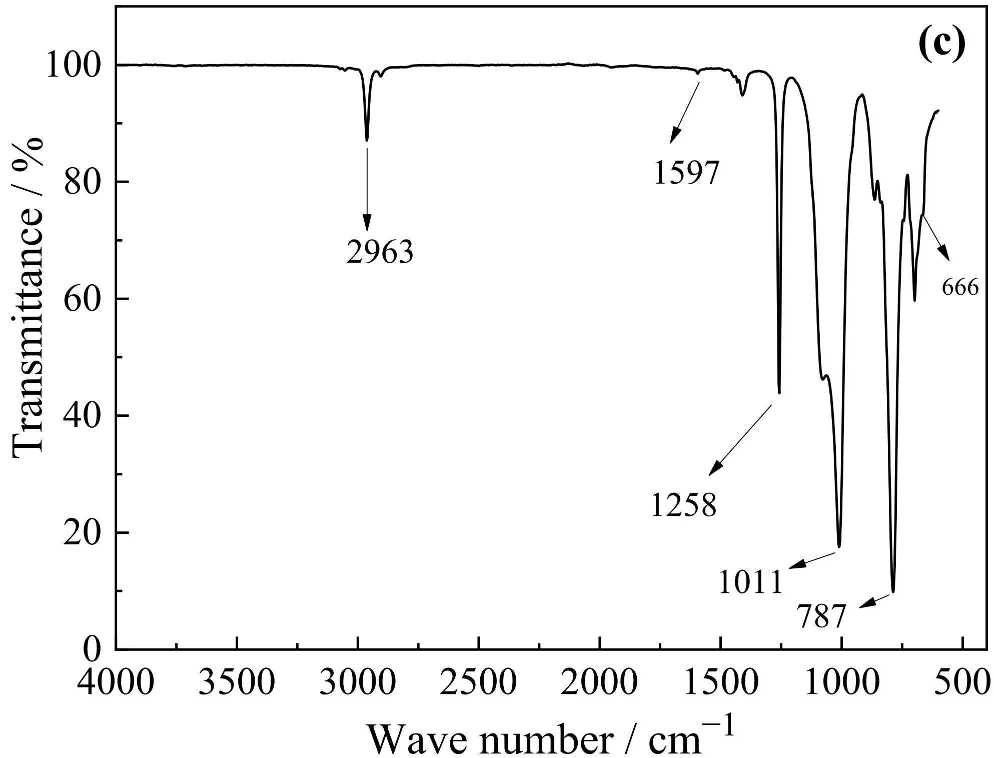
(c)
Transmittance / %
0
20
40
60
80
100
2963
1597
1258
1011
787
666
4000 3500 3000 2500 2000 1500 1000 500
Wave number / cm^{-1}

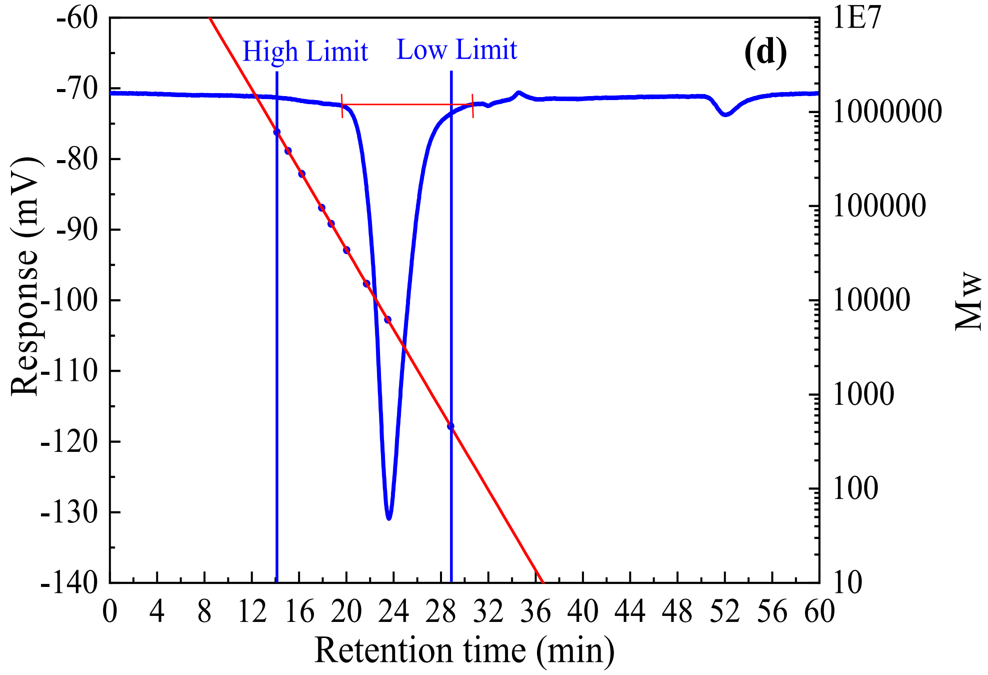

Figure S4. The ^{1}H NMR spectrum (a), ^{13}C NMR spectrum (b), FT-IR spectrum (c), and GPC curve (d) of Ph_2SiO bridged Si-Vi terminated linear siloxane oligomers (Ph_2SiO-SiVi) (Table S6, Entry Ph_2SiO-SiVi-B18), $CDCl_3$ was used as the solvent in NMR analysis.

Part III. Structural analysis of typical polymers

S3.1 Ph_2SiO bridged Si-H terminated linear siloxane oligomers (Ph_2SiO-SiH)

As shown in Figure S2a, δ=7.26 ppm was the chemical shift of the proton in C***D***Cl_3, δ=0.03~0.19 ppm were the chemical shifts of the protons attributed to -Si-C$\boldsymbol{H}_3$ located at side chain and terminal position in the polymer molecule, δ=1.50~1.70 ppm were the chemical shifts of protons in -Si-O***H*** or trace amounts of $\boldsymbol{H}_2$O in deuterated chloroform, δ=4.68~4.72 ppm were the chemical shifts of protons in -Si-***H***, and δ=7.30~7.75 ppm were the chemical shifts of all proton on the benzene ring in -Si-Ph. According to the structural formula and ^{1}H NMR spectrum of Entry Ph_2SiO-SiH-B18, there was only one proton on the Si-H bond; if its integrated area was defined as 1.00, the integrated area of all protons on -Si-C$\boldsymbol{H}_3$ was 115.51. From 6n+6=115.51, one can calculate that n≈18, and the molecular weight of the synthesized product is determined to be M_{NMR}=3002 g/mol.

From the ^{13}C NMR spectrum of the sample (Figure S2b), it could be seen that δ=76.75 ppm was the chemical shift of C atom in ***C***DCl3, δ=0~1.05 ppm were the chemical shift of C atoms in the terminal or sided ***C***H_3 connected to Si-O bonds, and δ=127.40~136.14 ppm were the chemical shift of C atoms in -Si-Ph. Due to the influence of the testing environment and the presence of benzene rings in the polymer, the intensity of the C atom peak became smaller, and the characteristic peaks were not pronounced, as shown in positions a and c in Figure S2b.

As shown in Figure S2c, δ=−6.93 ppm was the characteristic peak of Si atoms in ***Si***-H; at chemical shift δ=−21.95~−20.01 ppm, these signals were attributed to the characteristic peak of Si atoms in D-chain (-O-***Si***$(CH_3)_2$-O- segments), and at chemical shift δ=−48.49 ppm, the characteristic peak of Si atom attributed to D-chain ($-O_2$***Si***-Ph_2) could be clearly observed.

In the FT-IR spectrum shown in Figure S2d, the wave number at 2963 cm^{-1} was the C-H stretching vibration peak in -Si-CH_3; the wave number at 2127 cm^{-1} was the stretching vibration peak of -Si-H; the wave numbers at 1258 cm^{-1} and 787 cm^{-1} were the characteristic peaks of -Si-CH_3, the wave number at 1011 cm^{-1} was the

characteristic peak of Si-O-Si, and the wave number at 666 cm^{-1} was the characteristic peak of -Si-Ph.

In the GPC curve of the polymer sample (Figure S2e), only one sample peak was observed, which clearly conformed to the characteristics of high molecular weight polymers and showed a typical distribution characteristic. The weight average molecular weight (*M*w) of the measured sample was 4800 g/mol, the number average molecular weight (*M*n) was 2500 g/mol, and the polydispersity index (PDI) was about 1.87. Since the synthesized target product contained two phenyl groups, its molecular weight distribution was broad with a large PDI value.

S3.2 Si-Vi terminated T-shaped siloxane oligomers (PhT-SiVi)

As shown in Figure S3a, δ=7.26 ppm was the chemical shift of the proton in $C\underline{\boldsymbol{D}}Cl_3$, δ=0.05~0.18 ppm were the chemical shifts of the protons attributed to -Si-$C\underline{\boldsymbol{H}}_3$ located at side chain and terminal position in the polymer molecule, δ=1.50~1.70 ppm were the chemical shifts of protons in -Si-O$\underline{\boldsymbol{H}}$ or trace amounts of $\underline{\boldsymbol{H}}_2O$ in deuterated chloroform, δ=5.71~6.16 ppm were the chemical shifts of protons in -Si-$C\underline{\boldsymbol{H}}=C\underline{\boldsymbol{H}}_2$, and δ=7.30~7.80 ppm were the chemical shifts of all proton on the benzene ring in -Si-Ph. According to the structural formula and ^{1}H NMR spectrum of Entry PhT-SiVi-B18, there were three protons on the Si-$C\underline{\boldsymbol{H}}=C\underline{\boldsymbol{H}}_2$ group; if their integrated area was defined as 3.00, the integrated area of all protons on -Si-$C\underline{\boldsymbol{H}}_3$ was 116.83. From 6n+6=116.83, one can calculate that $n\approx19$, and the molecular weight of the synthesized product is determined to be M_{NMR}=4515 g/mol.

From the ^{13}C NMR spectrum of the sample (Figure S3b), it could be seen that δ=76.75 ppm was the chemical shift of C atom in $\underline{\boldsymbol{C}}DCl_3$, δ=0~1.16 ppm were the chemical shift of C atoms in the terminal or sided $\underline{\boldsymbol{C}}H_3$ connected to Si-O bonds, and δ=127.43~134.03 ppm and δ=139 ppm were the chemical shift of C atoms in -Si-Ph. The signals at δ=125 ppm and ca. δ=138 ppm were the chemical shifts of C atoms in -Si-$\underline{\boldsymbol{C}}H=\underline{\boldsymbol{C}}H_2$ group.

As shown in Figure S3c, δ=−4.11 ppm was the characteristic peak of Si atoms in $\underline{\boldsymbol{Si}}$-$CH=CH_2$; at chemical shift δ=−21.94~−20.91 ppm, these signals were attributed to the characteristic peak of Si atoms in D-chain (-O-$\underline{\boldsymbol{Si}}(CH_3)_2$-O- segments), and at

chemical shift δ=−80.85 ppm, the characteristic peak of Si atom attributed to T-segment ($-O_3$***Si***-Ph) could be clearly observed.

In the FT-IR spectrum shown in Figure S3d, the wave number at 2963 cm^{-1} was the C-H stretching vibration peak in $-Si-CH_3$; the wave number at 1597 cm^{-1} was the stretching vibration peak of $-Si-CH=CH_2$; the wave numbers at 1258 cm^{-1} and 787 cm^{-1} were the characteristic peaks of $-Si-CH_3$, the wave number at 1011 cm^{-1} was the characteristic peak of Si-O-Si, and the wave number at 666 cm^{-1} was the characteristic peak of -Si-Ph.

In the GPC curve of the polymer sample (Figure S3e), only one sample peak was observed, which clearly conformed to the characteristics of high molecular weight polymers and showed a typical distribution characteristic. The weight average molecular weight (Mw) of the measured sample was 5500 g/mol, the number average molecular weight (Mn) was 3300 g/mol, and the polydispersity index (PDI) was about 1.67. Since the synthesized target product contained one phenyl and has a T-type structure, its molecular weight distribution was wide with a large PDI value.

S3.3 Ph_2SiO bridged Si-Vi terminated linear siloxane oligomers (Ph_2SiO-SiVi)

As shown in Figure S4a, δ=7.26 ppm was the chemical shift of the proton in C***D***Cl_3, δ=0.04~0.17 ppm were the chemical shifts of the protons attributed to -Si-C***H***$_3$ located at side chain and terminal position in the polymer molecule, δ=1.50~1.70 ppm were the chemical shifts of protons in -Si-O***H*** or trace amounts of ***H***$_2$O in deuterated chloroform, δ=5.71~6.16 ppm were the chemical shifts of protons in -Si-C***H***=C***H***$_2$, and δ=7.30~7.80 ppm were the chemical shifts of all proton on the benzene ring in -Si-Ph. According to the structural formula and ^{1}H NMR spectrum of Entry Ph_2SiO-SiVi-B18, there were three protons on the Si-C***H***=C***H***$_2$ group; if its integrated area was defined as 3.00, the integrated area of all protons on -Si-C***H***3 was 143.38. From 6n+6=143.38, one can calculate that n≈23, and the molecular weight of the synthesized product was determined to be M_{NMR}=3777 g/mol.

From the ^{13}C NMR spectrum of the sample (Figure S4b), it could be seen that δ=76.75 ppm was the chemical shift of C atom in ***C***DCl_3, δ=0.97~1.17 ppm were the chemical shift of C atoms in the terminal or sided ***C***H_3 connected to Si-O bonds, and

δ=129.72~136.11 ppm were the chemical shift of C atoms in -Si-Ph. The signals at δ=127.55 ppm and ca. δ=139.36 ppm were the chemical shifts of C atoms in -Si-***C***H=***C***H_2 group.

In the FT-IR spectrum shown in Figure S4c, the wave number at 2963 cm^{-1} was the C-H stretching vibration peak in -Si-CH_3; the wave number at 1597 cm^{-1} was the stretching vibration peak of -Si-CH=CH_2; the wave numbers at 1258 cm^{-1} and 787 cm^{-1} were the characteristic peaks of -Si-CH_3, the wave number at 1011 cm^{-1} was the characteristic peak of Si-O-Si, and the wave number at 666 cm^{-1} was the characteristic peak of -Si-Ph.

In the GPC curve of the polymer sample (Figure S4d), only one sample peak was observed, which clearly conformed to the characteristics of high molecular weight polymers and showed a typical distribution characteristic. The weight average molecular weight (Mw) of the measured sample was 5700 g/mol, the number average molecular weight (Mn) was 3600 g/mol, and the polydispersity index (PDI) was about 1.58. Since the synthesized target product contained two phenyl groups, its molecular weight distribution was wide with a large PDI value.
